# The role of n-3-derived specialised pro-resolving mediators (SPMs) in microglial mitochondrial respiration and inflammation resolution in Alzheimer’s disease

**DOI:** 10.1186/s13024-025-00824-1

**Published:** 2025-03-21

**Authors:** Mary Slayo, Christoph Rummel, Pasindu Hansana Singhaarachchi, Martin Feldotto, Sarah J. Spencer

**Affiliations:** 1 School of Health and Biomedical Sciences, RMIT University, Bundoora, Melbourne, VIC Australia; 2 Institute of Veterinary Physiology and Biochemistry, Justus Liebig University Giessen, Giessen, Germany; 3 Center for Mind, Brain and Behavior - CMBB, Giessen, Marburg, Germany

**Keywords:** Alzheimer’s disease, Beta-oxidation, Fatty acids, Inflammation, Microglia, Mitochondria, N-3, Specialised pro-resolving mediators, Sex differences

## Abstract

Alzheimer’s disease (AD) is the most common form of dementia globally and is characterised by reduced mitochondrial respiration and cortical deposition of amyloid-β plaques and neurofibrillary tangles comprised of hyper-phosphorylated tau. Despite its characterisation more than 110 years ago, the mechanisms by which AD develops are still unclear. Dysregulation of microglial phagocytosis of amyloid-β may play a key role. Microglia are the major innate immune cell of the central nervous system and are critical responders to pro-inflammatory states. Typically, microglia react with a short-lived inflammatory response. However, a dysregulation in the resolution of this microglial response results in the chronic release of inflammatory mediators. This prolongs the state of neuroinflammation, likely contributing to the pathogenesis of AD. In addition, the microglial specialised pro-resolving mediator (SPM) contribution to phagocytosis of amyloid-β is dysregulated in AD. SPMs are derivatives of dietary n-3 polyunsaturated fatty acids (PUFAs) and potentially represent a strategic target for protection against AD progression. However, there is little understanding of how mitochondrial respiration in microglia may be sustained long term by n-3-derived SPMs, and how this affects their clearance of amyloid-β. Here, we re-evaluate the current literature on SPMs in AD and propose that SPMs may improve phagocytosis of amyloid-β by microglia as a result of sustained mitochondrial respiration and allowing a pro-resolution response.

## Introduction

Alzheimer’s disease (AD) is an age-related neurodegenerative disorder, estimated to cost Australia around $26.6 billion annually by 2041 [1] and to cost the US $1.1 trillion annually by 2050 [2]. The symptomatic presentation of AD is characterised by progressive memory loss and decline in cognitive function, accompanied by neurotoxic accumulation of amyloid-β plaques and intracellular hyperphosphorylated tau (p-tau) [3, 4]. In early-to-mid stages of AD (Braak stage I-IV), human AD brains show an increase in tau phosphorylation at Thr231, Ser199, and Tyr18 [5]. Stages I-II show early oxidative damage, dysfunction in energy production [6], and deficits in complex I of the mitochondrial respiratory chain [7], while stages III-IV denote the start of amyloid-β plaque formation [5]. In later stages (Braak stage V/VI), pathological changes reach their peak including deposition of amyloid-β plaques and phosphorylation of Thr231, Tyr18, Ser199, Ser202/Thr205 and Ser422 [5]. This is accompanied by defective mitophagy [8], decreased levels of mitochondria-related proteins [9], and decreased expression in mitochondrial complexes I, II, IV and V in human entorhinal cortex [10]. These dysregulations in mitochondrial activity may play a central role in this accumulation of amyloid-β and p-tau [11]. Mitochondrial dysregulation further contributes to all AD-associated pathologies by disrupting optimal cellular functioning and increasing cell death [12].

Mitochondrial dysregulation occurs in models of AD prior to plaque formation. For instance, the transgenic Thy-1 amyloid precursor protein (APP) mouse model of AD shows a concomitant drop in mitochondrial membrane potential and adenosine triphosphate (ATP) levels at 3 months of age alongside increased intracellular amyloid-β, events that precede extracellular plaque formation [13]. Intracellular and oligomeric forms of amyloid-β then lead to the formation of reactive oxygen species (ROS) and worsen dysfunctional mitochondrial and proteasomal functioning [14–16]. Data in humans suggest mitochondrial dysregulation is highly relevant to AD progression [17–20].

With early dysfunction in mitochondrial function being key to AD [13], it may be posited that one of the most important dysregulated pathways during AD pathogenesis is the metabolism of lipids to generate energy. The metabolites of various lipids support cell survival, proliferation, ATP production, and respiration in neurons and glia [21]. The growing field of lipidomics—the study of lipid metabolism pathways in biological systems—has provided insight into the role of lipid mediators in the immune system. Both n-3 and n-6 polyunsaturated fatty acids (PUFAs) are integral to brain functioning, becoming esterified into the phospholipid bilayer of neuronal and glial cell membranes where they maintain membrane function, and regulate gene expression and synthesis of lipid mediators [22]. N-3 and n-6 PUFAs comprise 20% and 10%, respectively, of total brain phospholipid composition [23]. During inflammation, n-3 and n-6 PUFAs are metabolised to allow transcellular biosynthesis of specialised pro-resolving mediators (SPMs) at local cells to respond to and resolve inflammation at an injury site [24]. These SPMs may be crucial to long-term mitochondrial metabolism and maintaining brain health.

Within microglia, SPMs have neuroprotective effects by acting as chemical messengers that allow microglia to morphologically and functionally respond to inflammatory stimuli preceding and during AD [25]. Arguably, this is a neuroprotective effect, with the pro-resolving cascade being largely due to SPM regulation of mitochondrial metabolism in microglia. Initially in response to a stimulus such as neuronal damage, the n-6 PUFA, arachidonic acid (AA), is metabolised to produce pro-inflammatory mediators including prostaglandins (PGs), leukotrienes, (LTs) and thromboxanes (TXs) that facilitate an inflammatory response from microglia and astrocytes [26, 27]. Lipoxins (LXs) are metabolised from n-6 AA lipoxins via lipoxygenases, while prostaglandins (PGs), leukotrienes (LTs) and thromboxanes (TXs) are metaoblised from n-6 AA via both lipoxygenases and cyclooxygenases [25]. Secondly, metabolism of n-3 PUFAs eicosapentaenoic acid (EPA) and docosahexaenoic acid (DHA) drives the synthesis of pro-resolving lipid mediators [24] (See Table 1). Maresins, neuroprotectins, and resolvins of the D series (RvD) are derived from DHA via catalysis from lipoxygenases, while resolvins from the E series (RvE) are generated from EPA through cyclooxygenases [28]. Once the toxic stimulus has been removed, the local secretion of RvE and RvD, PD, and MaR promotes a pro-resolving phenotype characterised by increased efferocytosis and a dampened inflammatory response [29–31]. For instance, the SPM n-3 derivative, lipoxin A4 (LXA4) has pro-resolving effects by reducing ROS production in pro-inflammatory BV2 microglia cells [32], and inhibiting interleukin (IL)−8 expression from 1321N1 human astrocytoma cell [33]. Together, this evidence suggests SPMs are crucial regulators of the microglial pro-resolving during inflammation.

**Table 1:** The pro-resolving and pro-inflammatory effects of specialised pro-resolving mediators

Specialised pro-resolving mediator(s)	Precursor	Effect	Action
Lipoxins (LXA4, LXB4)	Arachidonic acid (AA)	Pro-resolving	Downregulates IL-1β and TNF-α production [34] Promotes pro-resolving microglia phenotype [35]
Resolvins (RvD1, RvD2, RvD3, RvE1, RvE2)	Docosahexaenoic acid (DHA) and eicosapentaenoic acid (EPA)	Pro-resolving	Downregulates pro-inflammatory cytokine production [36] Promotes pro-resolving microglia phenotype [35] Increases phagocytosis of amyloid-β [36]
Protectins (PD1, PDX)	DHA	Pro-resolving	Promotes neuronal survival and induces anti-apoptotic gene expression [37] Promotes pro-resolving microglia phenotype [35]
Maresins (MaR1, MaR2)	DHA	Pro-resolving	Promotes pro-resolving microglia phenotype [35] Increases protein levels related to survival pathway, PI3K/AKT [38] Decreases inflammatory and apoptotic pathway expression including mTOR and caspase 3 [38]
Prostaglandins (D2, E2)	AA	Pro-inflammatory	Disruption of blood–brain-barrier [39] Promotes microglial pro-inflammatory phenotype [40] Increases neuropathic pain by modulating neuronal and microglia activity [41] Potential role in neurodegenerative diseases by exacerbating neuroinflammation [42]
Thromboxanes	AA	Pro-inflammatory	Increases platelet aggregation [43] Impairs integrity and function of blood–brain-barrier [44]
Leukotrienes (B4, C4)	AA	Pro-inflammatory	Promote leukocyte recruitment to the brain [45] Increases vascular permeability in the brain [46] Increases pro-inflammatory microglial phenotype [43]

In neurodegenerative diseases, these n-3 and n-6 derived SPMs show a clear relationship to neuropathological severity and stage. In preclinical models of Parkinson’s disease (PD), RvD1 inhibits the progression of neuropathology by supressing the microglial pro-inflammatory response and preventing motor impairments [47, 48]. In PD patients (*n* = 8), cerebrospinal fluid (CSF) and plasma levels of RvD1 are lower than the healthy controls (*n* = 8), with no differences in RvD2 [47]. Similar CSF patterns are seen in AD patients (*n* = 25) with lower CSF n-3 PUFA levels than individuals who were cognitively healthy with either no neuropathology (*n* = 36) or with neuropathology (*n* = 34) with no change in n-6 PUFAs across all groups [49]. Interestingly, each clinical group showed a distinct pattern of relationships between their CSF levels of amyloid-β_42_ and the ratio of n-3/n-6 PUFAs [49], demonstrating that these lipidomic profiles are disease-stage specific. With the newness of n-3 research in AD, current literature has not, to our knowledge, yet observed any distinction in the effects of RvE and RvD in AD, yet both seem to be beneficial in reducing amyloid-β pathology. When increasing RvD1 levels in mildly cognitive impaired (MCI) patients via n-3 supplementation, isolated macrophages in vitro show increased phagocytosis of amyloid-β [50]. Similarly, RvE1 treatment in 5xFAD mice reduces amyloid-β plaque deposition and release of inflammatory cytokines [51]. Together, these show that CSF and plasma n-3 derived SPMs have a clear relationship to and are crucial in responding to pathology during neurodegenerative diseases.

**Fig. 1:**
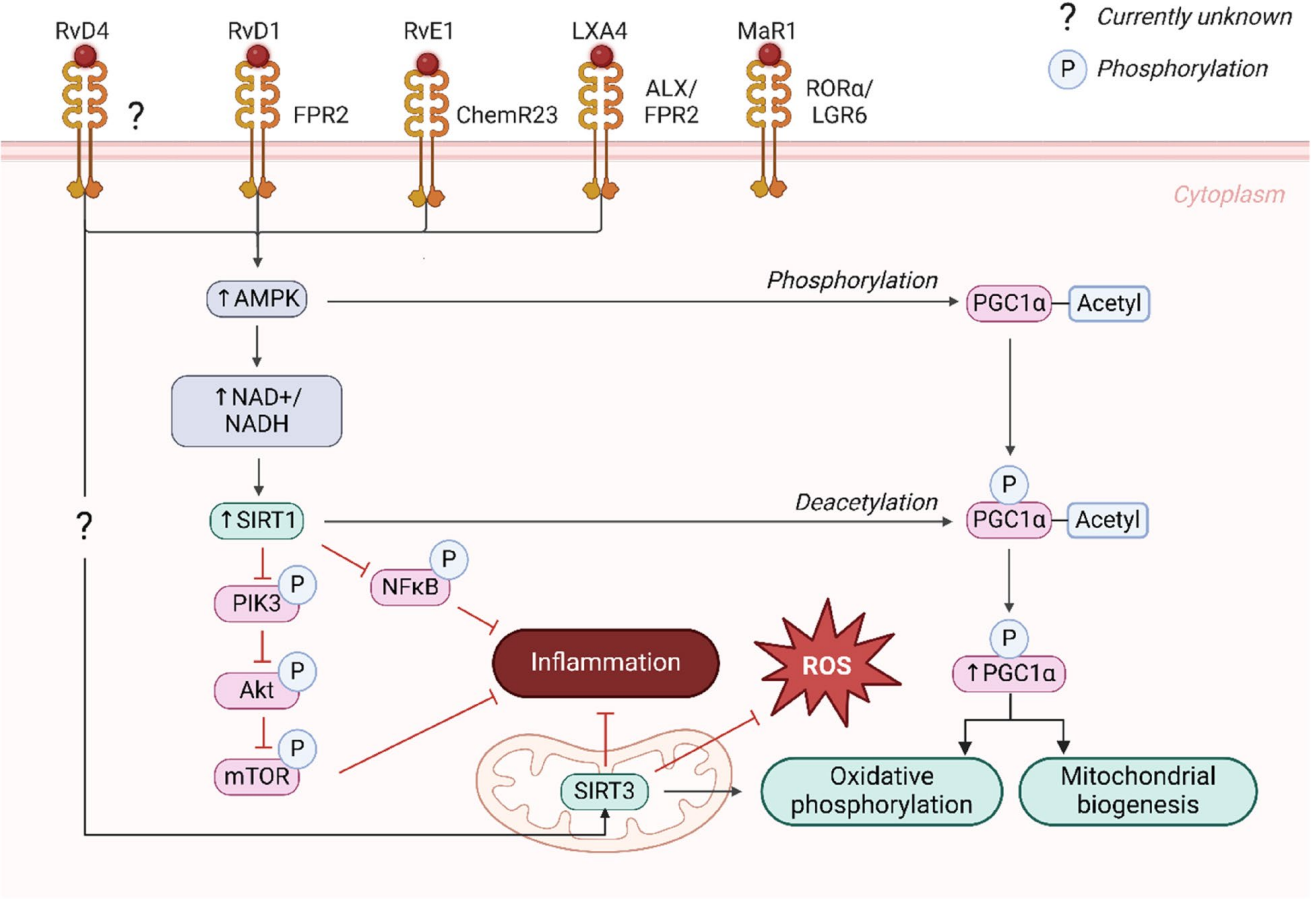
Current understanding of how specialised pro-resolving mediators (SPMs) enhance sirtuin levels and subsequent mitochondrial activity in cells. In the brain, SPMs activate their respective receptors and upregulate adenosine monophosphate-activated protein kinase (AMPK) activity. AMPK upregulation stimulates the activity of the peroxisome proliferator-activated receptor gamma coactivator 1-alpha (PGC-1α) via its phosphorylation and deacetylation. Currently, resolvin D4 (RvD4) is shown to increase sirtuin (SIRT)−3 activity and subsequent mitochondrial respiration, although its receptor and pathway is unknown. Simultaneously, AMPK upregulation increases activity of nicotinamide adenine dinucleotide (NAD) and NADH, which stimulates SIRT1, and inhibits the inflammatory pathways including phosphatidylinositol 3-kinase (PIK3), protein kinase B (Akt), and mammalian target of rapamycin (mTOR). Together, these lead to an increase in oxidative phosphorylation and mitochondrial biogenesis, and reduction in reactive oxygen species (ROS) release and activity of the inflammatory pathway. Rv: Resolvin; FPR2: N-formyl peptide receptor 2; ChemR23: chemerin receptor 23; ALX/FPR2: lipoxin receptor/N-formyl peptide receptor; MaR: maresin; RORα/LGR6: retinoic acid-related orphan receptor α/leucine-rich repeat domain-containing G protein-coupled receptor 6; PIK3: phosphoinositide 3-kinase; Akt: protein kinase B; mTOR: mammalian target of rapamycin. Figure created with BioRender.com; Toronto, Canada. Adapted from [53, 56–67]

While the direct mechanism of this SPM-mediated neuroprotection is unknown, it has been theorised to be related to improved mitochondria function and integrity [52]. Treating cells with RvD1 has proven effective in rescuing inflammation-driven mitochondrial damage, by restoring ATP production and repairing mitochondrial structural integrity [53]. Mechanistically, RvD1, RvE1, and MaR1 enhance mitochondrial respiration by increasing sirtuin 1 (Sirt1) via adenosine monophosphate-activated protein kinase (AMPK) signalling (Fig. 1) [53]. While research into the relationship between SPMs and mitochondrial bioenergetics in microglia specifically is scarce, research performed in other cell types provides an insight into the mechanisms and potential benefits of SPMs in AD-mediated mitochondrial damage in microglia [53–55]. Therefore, sufficient SPM bioavailability in the central nervous system (CNS) may allow for the restoration of tissue homeostasis by promoting a microglial pro-resolving phenotype that is mediated by sustained mitochondrial respiration. In this review, we will discuss the importance of microglia’s mitochondrial bioenergetics during neuroinflammation specifically in AD, the role of n-3-derived SPMs on microglia’s respiratory needs when responding to AD neurotoxicity, and the potential of using n-3-derived lipid metabolites for cellular respiration-based therapeutic strategies.

### Mitochondrial changes in Alzheimer’s disease

The mechanism triggering mitochondrial dysfunction is likely to be inflammatory. In models of sepsis, inflammation has detrimental effects on mitochondria, resulting in reduced ATP production and mitochondrial damage including swelling, fragmentation, and rarefaction of the cristae in proximal tubular cells [52]. In the context of AD, exposure of hippocampal neurons to amyloid-β reduces mitochondrial anterograde motility across axons [68]. This ultimately decreases ATP supply and leads to the degradation of the synapses required for signal transduction and cell survival [12]. These suboptimal brain health conditions are worsened by the high-energy demands of the resident brain immune cells, microglia, which become hyperactivated in response to the neuroinflammatory insult. Microglia challenged with synaptotoxic, soluble oligomers of amyloid-β engulf synapses even before plaques form yet blocking this process by inhibition of complement receptor CR3 rescues synaptic loss [69]. This hyperactivity may create a cycle of neuroinflammation, whereby the amyloid-β-mediated dysregulation of neuronal cellular metabolism causes an exacerbated inflammatory response in the microglia that is not only detrimental to surrounding cells but may also mask energy deficits of dysfunctional neurons and astrocytes. Stroke-affected brain regions with limited oxygen supply and rampant neuronal and astrocytic death have more *Iba1*-positive microglia concomitant with normal levels of glucose consumption [70]. However, as Backes et al. have shown [70], these metabolic levels then reduce to pathological concentrations when microglia disappear, showing the ability of microglia’s energy requirements to both increase during inflammatory insult and mask deficits from dying neurons and astrocytes.

Mitochondrial irregularities underpin many neurodegenerative diseases and contribute to cell death, including in AD [71]. The CNS has an incredibly high metabolic requirement, accounting for 20% of the total body’s metabolic expenditure, even at rest [72]. Adequate ATP production via oxidative phosphorylation by mitochondria is crucial to supply these energy needs and maintain proper CNS functioning. Interestingly, mitochondrial dysfunction and recycling of those mitochondria that are damaged show a stage-dependent pattern in humans [71]. In early sporadic AD (Braak stage II-III), human hippocampi show increased protein levels of the mitochondrial marker, PTEN-induced kinase 1 compared to age-matched controls. In later stages (Braak stage VI), an increase in Parkin RBR E3 ubiquitin protein ligase is seen [73]. These two enzymes are responsible for the tagging of damaged mitochondria to be recycled, suggesting that mitochondrial abnormalities begin early in disease and are stage-dependent [71]. Similar findings of stage-dependent mitochondrial dysfunction are seen in vivo*.* An 84-month longitudinal study of humans showing MCI demonstrated a steady decline in glucose metabolism across time in disease-associated brain regions [18]. Another study found that young carriers of the high-risk gene for AD, *APOE4*, demonstrate abnormally low rates of glucose metabolism in the brain in their 20 s—decades before dementia onset—a trend that is amplified across the development of MCI [18, 19]. This glucose hypometabolism corresponds to impaired synaptic functioning across temporoparietal and frontal brain regions and is associated with symptom severity in patients [17, 20, 74]. The effect is attributed to impaired mitochondrial energy metabolism [75]. Likewise, damaged neurons isolated from the posterior cingulate cortex of individuals with AD show decreased expression of nuclear genes encoding mitochondrial electronic transport chain (ETC) subunits compared to neurons isolated from the relatively spared primary visual cortex [76], reflecting a central pathological hallmark of dysfunctional mitochondrial respiration across AD progression. This finding not only exemplifies the strong link between metabolic respiratory dysfunction and AD pathogenesis, but also provides insights into possibilities for mitochondria metabolism-related proteins as blood biomarkers for AD.

### Microglia bioenergetics associated with pro-inflammatory and pro-resolving phenotypes in Alzheimer’s disease

Metabolism of n-3 PUFAs sustains microglial needs during various states of brain health and development and there can be significant consequences if not bioavailable [77]. In response to injuries of the CNS, the ATP requirement of microglia increases to allow the cells to undergo translocation, morphogenesis, and phagocytosis of cellular debris and damaged cells at an injury site [78]. This microglial pro-inflammatory response is accompanied by a switch to glycolytic metabolic pathways to meet the high-energy demands [79]. Glycolysis, despite being a less efficient mode of energy production (producing 15 times less ATP than oxidative phosphorylation) is the dominant metabolic pathway in pro-inflammatory immune cells [80]. Pro-inflammatory microglia use glycolysis for rapid surveillance and debris clearance, whilst pro-resolving phenotypes benefit from the sustained energy synthesis achieved by the oxidative phosphorylation pathway [81]. Stimulation of microglia triggers metabolic reprogramming towards increased anaerobic glycolysis and the pentose pathway oxidative branch while retaining mitochondrial activity [82]. For example, inhibiting the enzyme that regulates glucose metabolism—pyruvate dehydrogenase kinase 1—causes microglia to predominantly manifest an anti-inflammatory response [83]. Little is currently known about whether long-term changes in microglia bioenergetics can substantially reduce risk of developing AD. While research on microglial bioenergetics is expanding, the respiratory alterations underlying neurotoxic or protective microglia phenotypes are limited, especially in the context of sporadic AD. Current research assessing the effects of n-3 and their SPMs on glycolytic flux use cultured microglia or macrophages [53, 54, 57] and therefore lack contextual assessment of the neuroglial viability, hyper-sensitivity and dysfunctional changes that occur within an AD brain and contribute to microglial changes. Elucidating the link between inflammation and microglia energy metabolism in neurodegenerative conditions provides future opportunities to modulate microglia activity in response to chronic insults via their metabolism, which may improve neurocognitive outcomes in AD.

In microglia, pro-resolving phenotypes use oxidative phosphorylation as their main energy source [79], for which healthy mitochondria are essential. Following acute exposure to oligomeric and fibrillar amyloid-β, microglia can shift from oxidative phosphorylation to glycolysis through the mammalian target of rapamycin (mTor) pathway [84]. However, chronic inflammatory exposure reverses this effect, downregulating both glycolysis and oxidative phosphorylation and impairing microglial activity in response to subsequent inflammation [84]. This critical link between chronic inflammation in AD and mitochondrial respiration is exemplified in microglia of AD patients with *TREM-2* at-risk alleles and *Trem-2* knockout mice that exhibit dysfunctional glycolysis, reduced mTor signalling, and higher autophagy [85]. Trem-2 is a key modulator of mitochondrial respiration, yet its deficiency in AD patients and 5xFAD mouse models of AD enhances autophagy, impairs activation of the mTor pathway, and decreases glycolytic metabolites and tricarboxylic acid (TCA) cycle intermediates [85–87]. This has deleterious effects on AD progression, as microglia with reduced metabolic fitness and increased death cannot surround amyloid-β plaques as efficiently [88], impairing their ability to form a physical barrier to protect neurons. In mouse models of AD, areas of fluorescently labelled soluble amyloid-β_40_ and amyloid-β_42_ plaque hotspots with less microglia coverage have greater neuronal dystrophy [89]. Thus, reduced amyloid-β clearance by microglia in AD exacerbates neuronal dystrophy, illustrating the deleterious effects of impaired microglial respiration on global cerebral health. Surprisingly, Baik et al. [84] have shown that exposure to the cytokine, interferon gamma (IFN‐γ) restores glycolysis and activation of mTor in microglia, thereby rejuvenating cytokine secretion and phagocytosis in response to amyloid-β and improving disease symptoms in the 5XFAD mouse model of AD [84]. Thus, the importance of glycolysis cannot be understated, with its inhibition resulting in impaired microglial phagocytosis, ROS production and cytokine secretion at a time when a pro-inflammatory state is not only beneficial, but crucial to controlling disease progression [90]. Nonetheless, the pro-resolving phase in microglia relies on a timely switch towards oxidative phosphorylation [80], making SPM bioavailability integral to maintaining long-term brain health.

### Genetic alterations linking mitochondrial dysfunction and the microglia response in Alzheimer’s disease

Links between microglial respiratory function and the inflammatory response in AD are partially illuminated by gene set enrichment analyses, demonstrating how chronic changes to inflammatory pathways and mitochondrial ill-health go hand in hand. When examining altered pathways in AD, microglia-mediated immune responses and signalling of several metabolism-related enzymes are amongst the most affected [91]. These include phosphoinositide 3-kinases (*Pi3k*), protein kinase B (*Akt*), *mTor*, and mitogen-activated protein kinases (*MapK*) [91]. During respiration, upregulation of *PI3K* and *Akt* leads to activation of *mTor*, modulating cell activities including cell survival, growth, and cycle progression, thereby preventing apoptosis [92]. If *mTor* activation becomes dysfunctional, this results in greater cell death, creating a perpetual inflammatory state via the activity or death of microglia. However, many of the genes involved in the oxidative phosphorylation, glycolytic, and TCA cycle pathways are downregulated in AD [93]. For example, peroxisome proliferator-activated receptor-gamma coactivator-1-alpha, known as *Pgc-1α*, promotes mitochondrial biogenesis and induces more oxidative and less glycolytic metabolism [94]. Moreover, we previously reported that *Pgc-1α* mRNA-expression increases in the brain of aged compared to young rats but decreases during systemic inflammation [95]. Genome-wide microarray analyses show reduced levels of *PGC-1α* mRNA in the hippocampi of human post-mortem AD brains compared to healthy controls, and that PGC-1α protein levels are negatively associated with neurotic plaques and amyloid-β_1–42_ content [96]. Similarly, microglia isolated from APP/PS1 mice brains show an accumulation of damaged mitochondria and defective mitophagy, leading to impaired phagocytosis of amyloid-β [97]. Furthermore, the same paper found that supplementing APP/PS1 mice with urolithin A, a compound that induces mitophagy, enhanced the phagocytic efficiency of microglia and mitigated NLRP3/caspase-1-dependent neuroinflammation [97]. Taken together, these findings support the novel hypothesis that early and long-term maintenance of mitochondrial function can sustain the microglial phagocytic response to promote tissue homeostasis and deter chronic low-grade inflammation due to amyloid-β plaques and neuronal debris.

**Fig. 2:**
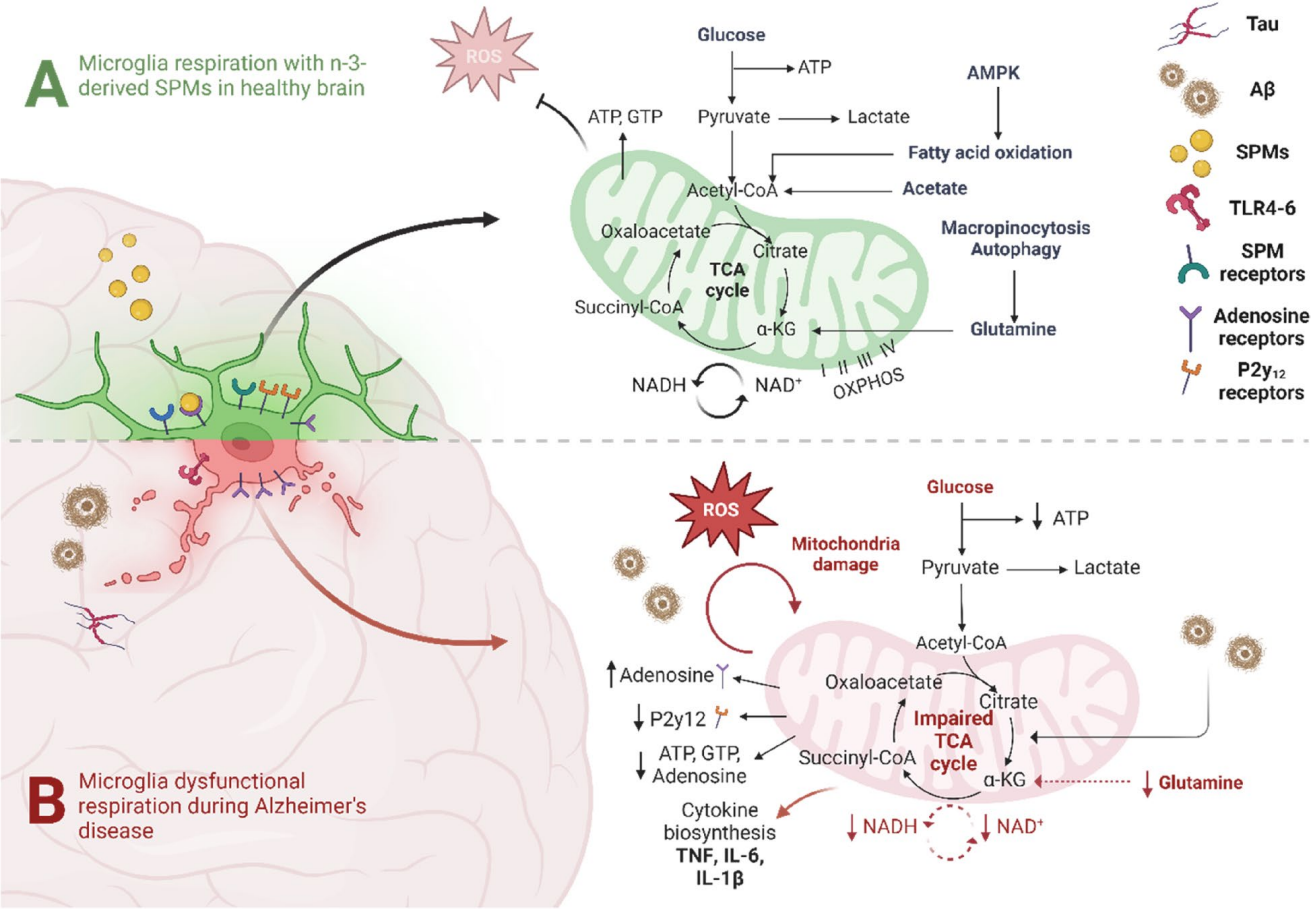
Microglial mitochondrial respiration in Alzheimer’s disease (AD). In the healthy brain, sufficient n-3 derived specialised pro-resolving mediators (SPMs) support fatty acid oxidation and oxidative phosphorylation to produce enough adenosine triphosphate (ATP) to meet metabolic demands and prevent excessive production of reactive oxygen species (ROS). This helps drive microglia towards a pro-resolving or homeostatic phenotype. In the AD brain, inadequate SPM action leads to a pro-inflammatory phenotype with reduced ATP production, increased cytokine synthesis and increased ROS production. The impairment in microglia viability and mitochondrial function reduces their ability to respond to stimuli such as amyloid-β (Aβ) and creates an excessively pro-inflammatory environment. TLR: toll-like receptor; TCA: tricarboxylic acid; NAD: nicotinamide adenine dinucleotide; TNF: tumor necrosis factor; IL: interleukin; α-KG: α-ketoglutarate; AMPK: AMP-activated protein kinase; GTP: guanosine triphosphate. Figure created with BioRender.com; Toronto, Canada. Adapted from [75, 136]

The failure of inflammatory resolution in AD may therefore be largely underpinned by dysregulated microglial oxidative phosphorylation, preventing the microglia from adopting a pro-resolving phenotype. Bioinformatics and gene set enrichment analyses consistently identify down-regulation of markers associated with mitochondrial oxidative phosphorylation as a hallmark of AD [98, 99]. One such newly discovered mutation that increases the risk of AD, *SHMOOSE*, is a mitochondria-encoded gene associated [100]. Assessing CSF samples from 79 individuals without dementia, *SHMOOSE* levels were positively associated with atrophy in medial temporal brain regions, as well as CSF total tau and p-tau 181. This finding was supported by RNA expression in the temporal cortex of 82 post-mortem AD brains, showing 15% greater expression of *SHMOOSE* RNA than healthy controls. Similar studies of proteomics performed on the cortices of AD patients identified that proteins in the oxidative phosphorylation pathway are amongst the most under-expressed in AD [101]. Additionally, nuclear-encoded, but not mitochondria-encoded, genes for oxidative phosphorylation are downregulated in the hippocampi of AD patients yet, curiously, the same genes are upregulated in patients with MCI [102]. This increase in patients with MCI could be representative of an early reparative attempt. Compared to post-mortem brains of those with late AD, those with early AD show downregulated expression of genes involved in complex I of the oxidative phosphorylation pathway, while complexes III and IV are upregulated [7]. These AD-induced changes in enzymatic activity in the TCA cycle follow a distinct pattern that is linked to clinical state, with the dehydrogenases/decarboxylases (including pyruvate dehydrogenase complex, isocitrate dehydrogenases, and the alpha-ketoglutarate dehydrogenase complex) being reduced while dehydrogenases (including succinate dehydrogenase and malate dehydrogenase) are increased [103] (See Table 2 for more related genes). There is also evidence of AD-related changes in other key molecular players involved in the energy-synthesis pathway. This includes lower levels of free guanosine triphosphate [104] as well as reduced microglial expression of the adenosine diphosphate receptor, P2y12 [105, 106]. Concomitantly, there is an early increase in cortical adenosine 1 (A_1_) and A_2A_ receptors [107] but decrease in adenosine levels [108], suggesting a robust and stage-dependent dysfunction in energy charge. Ultimately, the mitochondriomes in AD brains are distinct from those in normal aging, suggesting that the progression of AD may be at least partially driven by dysregulation in mitochondrial complexes associated with aerobic respiration (e.g. ETC complexes and ATP-synthase) [109] (See Fig. 2 for summary). This brings us to two under-explored but exciting potentials: 1) that upregulating and sustaining oxidative phosphorylation via SPM production and secretion may dampen the neurotoxic effects of amyloid-β_42_ plaque formation and cellular death, and 2) the potential identification of blood biomarkers of proteins resultant from mitochondrial oxidative abnormalities using metabolomic approaches in peripheral cells in AD. However, chronic pro-inflammatory microglia are but one part in a cascade of inflammaging, of which may result from a multitude of aging-related changes across the lifespan. Further research is needed to understand whether a) pushing microglia bioenergetics towards greater oxidative phosphorylation via SPM bioavailability across the lifespan reduces the occurrence of the phenotypic aging-related pro-inflammatory microglia in later life, and b) subsequently reduces risk of AD.

**Table 2:** Common genetic alterations in Alzheimer’s disease that are linked to mitochondrial dysfunction

**Metabolic pathway**	**Gene name**	**Gene symbol**	**Direction of change**^**a**^	**References**	
				***Preclinical***	***Clinical***
FAO	NAD-dependent deacetylase sirtuin-1	SIRT1	Downregulated	*APPswe/PS1dE9 mice,* Lopes et al. [110] *APP/PS1 mice,* Li et al. [111]	Hadar et al. [112] Julien et al. [113]
FAO/CP	NAD-dependent deacetylase sirtuin-2	SIRT2	Upregulated	*SH-SY5Y cells,* Silva et al. [114] *APP/PS1 mice,* Bai et al. [115]	Minjarez et al. [101] Wongchitrat et al. [116]
FAO	Peroxisome proliferator–activated receptor γ coactivator 1α	PGC-1α	Downregulated	*Tg2576 neurons,* Qin et al. [96] *APP/PS1 mice,* Shi et al. [117] *2xTg-AD mice,* Wang et al. [118]	Qin et al. [96] Katsouri et al. [119]
OXPHOS	NADH dehydrogenases subunits 4 and 8	NDUFS4, NDUFS8	Downregulated	*TgCRND8 mice,* Francis et al. [120] *Ndufs4*^*−/−*^* mice* Gao et al. [121]	Brooks et al. [93] Mastroeni et al. [102] Adav et al. [109] Lunnon et al. [122]
OXPHOS	Ubiquinol-cytochrome *c* reductase core protein II	UQCRC2	Downregulated	*3xTg AD mice*, Xie et al. [123] *TgF344-AD rats,* Rudisch et al.[124]	Brooks et al. [93] Mastroeni et al. [102] Adav et al. [109] Lunnon et al. [122]
OXPHOS	Cytochrome *c* oxidase subunit IV isoform 1 and subunit VIa polypeptide 1	COX4I1, COX6A1	Downregulated	*HO_TASTPM mice,* Bi et al. [125] *Tg2576 mice,* Morello et al. [126]	Brooks et al. [93] Mastroeni et al. [102] Lunnon et al. [122]
OXPHOS	ATP synthase, F1 subunit beta	ATP5B	Downregulated	*Presenilin 1 familial Alzheimer’s disease iPSC-derived neural stem cells,* Martín-Maestro et al. [127] *N2a neuroblastoma cells expressing the ApoE4 allele,* Orr et al. [128]	Brooks et al. [93] Mastroeni et al. [102] Adav et al. [109] Lunnon et al. [122]
Glycolysis	Glucose-6-phosphate isomerase	GPI/G6P	Downregulated	*APP/PS1 mice,* González-Domínguez et al. [129]	Saito et al. [130] Brooks et al. [93] Qiu et al. [131]
Glycolysis	Lactate dehydrogenase A	LDHA	Downregulated	*APP/PS1 mice,* Zhang et al. [132] *Tg2576 mice,* Morello et al. [126]	Niccoli et al. [133] Brooks et al. [93] Qiu et al. [131]
TCA	Malate dehydrogenase 1	MDH1	Downregulated	*Amyloid-β injected Wistar rats,* Shaerzadeh et al. [134] *APP/PS1 mice*, Correas et al. [135]	Jia et al. [209] Brooks et al. [93] Qiu et al. [131]

Direction of fold change compared to healthy controls^a^. While these genes are commonly identified as dysregulated in Alzheimer’s disease, they do not cover all alterations identified across the literature. *FAO* fatty acid oxidation, *CP* cell proliferation, *OXPHOS* oxidative phosphorylation

### How n-3-derived specialised pro-resolving mediators may rescue mitochondrial respiration via Sirtuin 1

In the search to examine how cellular respiration changes across AD pathogenesis, recent studies have identified a reduction in an important deacetylase responsible for mitochondrial biogenesis. SIRT1 is a NAD-dependent deacetylase that also functions as an energy sensor [137]. SIRT1 regulates lipid metabolism in the liver during energy deprivation via deacetylation of peroxisome proliferator-activated receptor-alpha (PPAR-α), supporting fatty acid β-oxidation and hence maintaining mitochondrial respiration [137]. Analysis of post-mortem temporoparietal regions of AD brains show significantly lower SIRT1 concentrations than cognitively healthy individuals [138]. This supports earlier research that found significantly reduced *SIRT1* mRNA and SIRT1 protein levels in the parietal cortex of AD patients that correlated with amyloid-β and tau pathogenesis [139]. In turn, several recent studies have identified Sirt1 signalling as the mechanism that improves cognitive deficits and reduces amyloid-β burden in transgenic mouse models of AD [140, 141]. Collectively, this suggests that Sirt1’s influence over cellular respiration may be a key player driving AD progression.

A reduction in Sirt1 has severe knock-on effects for the metabolism of n-3 PUFAs and biosynthesis of SPMs. Quantitative proteomics identifies that the key molecules altered by *Sirt1* depletion fall into functional categories including secreted factors (e.g. adiponectin, IL-1 receptor antagonist), and enzymes linked to lipid metabolism [142]. Specifically, fatty acid synthase (FAS) is among the enzymes altered by *Sirt1* depletion, and this plays a crucial role in the desaturase/elongase pathway to form PUFAs [143]. Knockdown of *Sirt1* reduces the n-3 PUFA metabolite, DHA’s, ability to deacetylate nuclear factor kappa B (NF-κB) in macrophages, worsening inflammation by increasing expression of tumor necrosis factor, IL-1β and IL-4 [144]. This is supported by a similar study that found that not only does DHA mimic the effects of Sirt1 on deacetylation of the NF-κB subunit p65, but DHA’s deacetylation and inhibition of downstream cytokine expression requires Sirt1 [54]. Thus, Sirt1 is a key mediator of the β-oxidation of fatty acids, resulting mitochondrial function, and suppression of cytokine transcription factors [54]. Interestingly, *SIRT1* and *SIRT3* have been detected in human serum, and while healthy aging individuals show reduced serum *SIRT1* levels compared to young healthy individuals, this decrease is significantly worsened in AD patients [145]. This provides an exciting opportunity to develop *SIRT1* as a predictive blood-based biomarker for the early stages of AD with known reference ranges that are distinct to healthy aging. Understanding of the role of SIRT1 in AD has also led to recent investigations into the effects of oral administration of the selective serotonin re-uptake inhibitor, alaproclate (A03), in mouse models of AD. In vivo A03 treatment not only increased *Sirt1* levels in hippocampi of 5xFAD mice, but also improved memory recall and showed no toxicity [146]. These studies show excellent potential for Sirt1-targetted therapeutics, including DHA with its stimulative effect on Sirt1 [147], for individuals with MCI to halt progression of AD-like pathology.

### Specialised pro-resolving mediators and Sirtuin 1 in mitochondrial respiration in microglia

Excitingly, n-3-derived SPMs increase mitochondrial respiration by increasing AMPK signalling and upregulating Sirt1 expression. While clinical data into the relationship between SPMs and SIRT1 expression is limited, microglia culture studies provide a glimpse into the mechanisms linking Sirt1, SPMs and the microglial inflammatory response. During lipopolysaccharide (LPS)-induced inflammation, microglia pre-treated with EPA and DHA show upregulated Sirt1 mRNA levels and Sirt1 protein deacetylase activity, as well as increased mRNA levels of nicotinamide phosphoribosyltransferase (*Nampt*) and thus NAD + within microglia [57]. However, when α1AMPK is knocked down, the ability for DHA to enhance Sirt1 levels in macrophages is blunted [54], suggesting that the ability for SPMs to upregulate mitochondrial respiration via Sirt1 requires AMPK. RvD1, RvE1 and MaR1 also enhance mitochondrial respiration through an AMPK-dependent signalling mechanism in macrophages [53]. This was also seen following LPS-induced inflammation where maresin conjugates in tissue regeneration 1 (MCTR1) rescues mitochondrial function by promoting activation of the receptors involved in mitochondrial biosynthesis, Pgc-1α and Sirt1 [55]. Exercise, another known stimulatory mechanism for pro-resolving SPM biosynthesis and catabolism of inflammatory lipid mediators, also enhances mitochondrial respiration in macrophages via the same AMPK signalling pathway that increases *Sirt1* [53].

Taken together, these data highlight the protective role for SPM-induced *Sirt1* signalling against inflammation and may be the same mechanism that can sustain a pro-resolving phenotype of microglia in the chronic low-grade systemic inflammation seen with aging –inflammaging—that precedes AD pathogenesis. Thus, maintaining mitochondrial respiration through the *AMPK/SIRT1* pathway could be necessary in maintaining long-term microglial respiration and phagocytic ability and reducing the pro-inflammatory response, and may be achieved by long-term bioavailability of SPMs. Having said this, there is currently very limited research into the rescue effect of Sirt1 signalling on chronically pro-inflammatory microglial responses in the context of the AD brain, and many considerations are warranted. Most studies have predominantly utilised isolated and cultured macrophage cell lines, wherein standardised laboratory procedures measuring mitochondrial activity at 37 degrees Celsius do not account for patho-physiological changes in body temperature. A recent in vitro study comparing mitochondria respiration in peripheral blood mononuclear cells (PBMCs) from healthy and depressed patients demonstrate that respiration differences disappear when accounting for higher body temperature of depressed individuals [148]. Similarly, while data from animal models of AD have shown that DHAs improve mitochondrial respiration [149], these studies have not been cell-specific and often do not assess the different roles of each SPM. It is important to consider that SPMs are generated at much smaller quantities than classical pro-inflammatory oxylipins like leukotrienes or prostaglandins and often exhibit volatile properties (as reviewed in [150]). Therefore, valid detection and quantification of SPMs represent a challenging endeavour. Ultimately, the ability of SPMs to rescue microglial mitochondrial respiration via *Sirt1* and any subsequent effects on amyloid-β clearance remain to be tested. In this light, future research should assess SIRT1 changes in the human brain across healthy, MCI, and late AD stages, and whether long-term bioavailability of n-3-derived SPMs can mediate microglial respiration and phagocytosis in response to stimuli in the inflammaging brain via SIRT1 activity.

### Sex differences in mitochondrial bioenergetics and risk of Alzheimer’s disease

Human sex differences in mitochondria metabolism with aging are notable and may partially account for women at 65-years-old having a 12% lifetime risk of AD compared to 6.3% for men [151]. Machine-learning analysis of brain positron emission tomography (PET) images from healthy men and women aged 20 to 82 years old found that aged female brains have a younger metabolic brain age than aged male brains, based on regional glucose levels, oxygen consumption, and cerebral blood flow [152]. Interestingly, the cerebral glucose levels in females accounted largely for these observed differences [152]. Similar findings of sex-based mitochondrial differences are observed in healthy adult women, where peripheral mononuclear blood cells show higher mitochondria complexes I, I + II, and IV, uncoupled respiration, ETC capacity, and ATP levels compared to men [153]. Similarly, in vivo markers of oxidative stress are higher in young, healthy men compared to premenopausal women when controlling for age, blood pressure, plasma cholesterol and glucose [154]. Estrogen is theorised to play a neuroprotective effect in this, largely due to its capacity to increase mitochondrial biogenesis and respiration in neurons and glia as well to reduce lipid peroxides [155]. Estrogen is known to increase expression of *Pgc-1a*, the transcription factor controlling energy, metabolic, mitochondrial function and biogenesis as previously discussed [156, 157]. To investigate the role of estrogen, a recent human neuroimaging study found post-menopausal women to have glucose hypometabolism in parietotemporal regions implicated in AD pathology, suggesting that reduced estrogen may play a role in cerebral metabolic dysregulation. However, the same study found post-menopausal women to have increased cerebral blood flow and ATP production relative to pre-menopausal women, with the ATP production positively correlating with cognitive performance [158]. This suggests a potential compensatory attempt, creating a new baseline in the post-menopausal female brain that largely mediates the onset of disease risk.

Animal models of AD have also been used to investigate the neuroprotective effects of estrogen on mitochondria and microglial function yet have so far failed to reach a consensus on its effect. In the 3xTg mouse model of AD, ovariectomy reduces mitochondrial respiration and increases amyloid-β levels in mitochondria and these amyloid-β levels are reduced again with estrogen treatment [159, 160]. This effect may be mediated by estrogen’s antioxidant activity, as estradiol replacement reduces hyperphosphorylation of tau in ovariectomised female hTau mice treated with amyloid-β_42_ compared to ovariectomised and sham females without estradiol [161]. Similar studies using microglia isolated from APP/PS1 mouse models show female microglia shift to glycolysis in the presence of amyloid-β plaques while male microglia do not [162]. Nonetheless, when the same researchers assessed post-mortem AD brains from human women, the microglia were more complex yet associated with increased plaque area while microglia from men with AD were more amoeboid and showed reduced plaque load [162]. Thus, while sex differences in cerebral metabolism are observed, their effect on the microglial response to amyloid-β and p-tau in AD remains unclear. Taken together, it can be predicted that a reduction in estrogen following menopause would negate any pre-menopausal metabolic protective effects. However, further investigation is needed into metabolic changes in the female brain post-menopause to better understand estrogen’s regulation of neuronal and glial metabolism, how these mediate neurodegenerative vulnerability in later life, and how metabolism-related preventative measures for AD can be tailored for peri-menopausal women.

With mitochondrial deoxyribonucleic acid (DNA) widely accepted to be inherited from mothers [163], there is even growing evidence of an inherited maternal link to AD. Cognitively normal, middle-aged children with AD-affected mothers, but not fathers, show lower cerebral glucose utilisation using fluorodeoxyglucose PET [164]. Subsequent studies of children from AD-affected mothers show greater degrees of cortical atrophy at a faster rate, greater amyloid-β plaque deposition, and greater levels of oxidative stress markers in CSF than those with paternal or no family history of AD [165–167]. These findings suggest that a) the mitochondrial genome and its function play a significant role in AD and that b) there is at least some maternally inherited component towards brain bioenergetic failure. However, the extent of the maternal inheritance, the mitochondrial genes involved in this component, and the epigenetic role of long-term maternal n-3 consumption on this component are yet to be determined.

### Clinical studies, implications and treatment approaches

Despite the importance of these mitochondrial respiratory pathways and the promise of SPM-mediated neuroprotection in pre-clinical studies, it is still not clear if n-3 supplementation can be beneficial in the clinic or whether any effects are attributed to SPMs and mitochondria function. One cross-sectional study assessing 320 cognitively unimpaired participants at increased risk of AD dementia measured the relationship between blood levels of n-3 alpha-linolenic acid (ALA) and DHA to brain glucose uptake [168]. Those with increased genetic risk (*APOE* ε4 load) exhibited a direct relationship between blood ALA and glucose uptake in vulnerable brain regions [168]. For DHA, direct associations were limited to those in the preclinical stage of AD pathology (positive amyloid-β and tau pathology) [168]. Most clinical studies to date have focused on cognitive function following n-3 consumption in MCI or AD patients, well after the chronic inflammatory cascade has begun [169]. At these stages, n-3 consumption will not halt cognitive decline but may slow it down. Indeed, an early clinical trial investigating n-3 PUFA effects on AD patients found no significant improvements in cognitive performance following 6-month supplementation with n-3 compared to control AD patients [169]. Nevertheless, the same study found n-3 supplementation halted cognitive decline across 6 months in those with very mild cognitive dysfunction. A more recent large clinical study using 211,094 individuals aged over 60 from the UK Biobank cohorts also found no significant association between fish oil supplementation and AD development [170]. Crucially, here, participants were simply classified as users or non-users at baseline testing, with no information on the formulation, dose, or duration of intake. Studies focusing on dietary n-3 as a risk-reduction rather than treatment of AD show greater promise. A 2023 analysis of 1,135 healthy participants prior to a dementia diagnosis (mean age = 73 years) from the Alzheimer's Disease Neuroimaging Initiative (ADNI) cohort assessed associations between long-term n-3 supplementation and AD incidence during a 6-year follow up. Long-term users demonstrated a staggering 64% reduction in risk of AD. These data were supported by a large meta-analysis on the relationship of n-3 and blood-based biomarkers to dementia risk, suggesting n-3 intake reduces total dementia risk by 20% [171]. Most recently, the DO-HEALTH trial (*n* = 777) demonstrated that 1 g/day of n-3 can slow biological aging by 2.9 to 3.8 months across 3 years, showing its potential for reducing risk of aging-related neurodegenerative disorders [172]. Thus, the relationship between n-3 consumption and risk of AD requires closer examination but is not without promise.

While clinical studies have assessed the effects of dietary n-3 on human cognition, almost none have distinctly provided SPMs as a treatment approach and assessed their therapeutic potential in reducing risk of AD. This includes pre- and post-treatment assessments with n-3-derived SPMs in MCI or AD patients, and whether these metabolites increase mitochondrial respiration in humans. While cognitive testing in clinical studies is sparse, preclinical mouse models using a cross-sectional design show promise for the neuroprotective effects of SPM treatment. In transgenic mouse models of AD, such as the Tg2576 model, LXA4 reduces amyloid-β levels and improves cognition [173]. Using the *App*^*NL−G−F/NL−G−F*^ mouse model, intranasal injection of a mix of SPMs (RvE1, RvD1, RvD2, MaR1, and NPD1) rescued memory deficits when compared to non-treated transgenic mice [174]. In a similar study using intrahippocampal injections of amyloid-β_42_ in mice, intracerebroventricular administration of MaR1 reduced cognitive decline compared to those only treated with amyloid-β_42_ [175]. It should be acknowledged preclinical studies are limited in translational value, as transgenic and injectable models of AD do not reflect the complexity of human AD pathogenesis [176]. This makes it difficult to predict if greater life-long dietary inclusion of SPMs would translate to reduced AD onset in the real world. The clinical studies showing neuroprotection of SPMs on cognition appear promising, yet it is unclear whether these are due to their pro-resolving SPM metabolites influencing mitochondrial respiration and whether these are enough to ameliorate cognitive decline. To assess this, clinical studies using a longitudinal, repeated measures design in aging populations are needed.

### Early diagnosis and disease progression using blood specialised pro-resolving mediators and mitochondrial-related genes

While limited in number, several recent clinical studies have shown that blood and CSF SPM levels are significantly reduced and can predict cognitive performance in patients with MCI and AD. One study assessing individuals with AD (*n* = 15), MCI (*n* = 20) and subjective cognitive impairment (*n* = 21) found a significant positive correlation between mini-mental state examination (MMSE) scores and CSF levels of LXA4 and RvD1 (Spearman’s Rho, *r* = 0.475, *p* < 0.0005 for LXA4 and *r* = 0.343, *p* < 0.05 for RvD1) [177]. This suggests a concentration-dependent SPM-mediated protection of memory function. Another study analysing CSF samples from 136 subjectively cognitively impaired (SCI), 43 MCI and 40 AD humans found lower levels of pro-resolving mediators RvD4, RvD1, NPD1, MaR1 and RvE4 in AD and MCI compared to SCI [178]. Further, AD and MCI patients had greater levels of the pro-inflammatory mediators leukotriene B_4_ and 15-hydroxyeicosatetraenoic acid (15-HETE) than healthy controls. In the AD patients, several lipid precursors of SPMs positively predicted MMSE scores including DHA, 14-hydroxy DHA, EPA, and AA [178]. For SCI patients, similar findings were observed with RvD1, RvD4 and MMSE scores [178]. While limited in number, these studies show potential for blood SPMs as early diagnostic biomarkers for MCI and AD, yet further research is needed to assess the replicability of these findings and if this relationship exists in presymptomatic humans.

Besides SPMs, many studies have used the early hypometabolic brain changes observed in AD to their advantage when assessing blood biomarkers related to cellular respiration. Sang et al. [179] have shown that glucose hypometabolism in the frontal, parietal, and temporal cortices of AD patients is associated with lower blood levels of thiamine diphosphate (TDP), a coenzyme of pyruvate dehydrogenase (PDHC) and α-ketoglutarate dehydrogenase (KGDHC) in the Krebs cycle and transketolase in the pentose phosphate pathway [179]. Genes related to oxidative phosphorylation are also differentially expressed in AD blood. Comparing blood from MCI (*n* = 168), AD (*n* = 164), and healthy individuals (*n* = 177), Lunnon and colleagues (2017) observed a concomitant decrease in expression of nuclear-encoded oxidative phosphorylation genes and increase in mitochondrial-encoded oxidative phosphorylation genes in MCI and AD patients compared to healthy controls [122]. Further, the pattern was notably worse in AD than MCI [122], suggesting selective block in their genetic translation that is stage dependent. It should be noted that the robustness of mitochondrial-related blood biomarkers also extends to type 2 diabetes; a condition that significantly increases risk of AD [180]. In type 2 diabetic patients with progressive MCI or AD, plasma levels of two neuroexosome-derived mitochondrial proteins—NADH ubiquinone oxidoreductase core subunit S3 (NDUFS3) and succinate dehydrogenase complex subunit B (SDHB)—were lower than in cognitively normal patients [181]. Together, these studies show a promising relationship between the earliest symptomatic stages of AD and blood levels of mitochondrial gene and protein expression.

### Methodological and clinical considerations

Despite these promising indications, we also note that considerations must made on the sensitivity of detecting and quantifying SPMs in biological tissue, and how this can limit its development as a clinical diagnostic tool. The most commonly accepted method of SPM quantification is liquid chromatography with tandem mass spectrometry (LC–MS-MS) including in preclinical rodents models [53] and human serum and plasma [182]. With this method, differences in levels of human SPMs have been reported between sex [183], age [184], and disease state [185]. However, a comprehensive review by Calder [185] reported on the number of studies and their detected concentration range of resolvins D and E series mediators in human plasma [185]. In most studies, the concentration was extremely low (< 50 pg/ml), close to the detection limit of several quantification methods [185]. Consistent with the low detection sensitivity, several studies have failed to detect SPMs in human plasma [186, 187], making them difficult to use as non-invasive biomarkers of inflammation in the clinic. Hence, reliability and sensitivity of SPM detection and quantification methods has been fundamentally questioned [188], and a consensus is yet to be determined before translation to the clinic can be considered.

Furthermore, we note that the consumption of n-3 should be balanced with its potential for neurotoxicity in large doses. For example, we have shown that a high n-3 diet in pregnancy can be toxic to foetal metabolic development outcomes in rats (1.66% total n-3) [189]. In humans, dietary n-3 decreases blood clotting (640 mg n-3 PUFAs for 4 weeks) [190] and blood pressure (3 g/day n-3 for 8 weeks) [191], which may pose a health risk for those with low blood pressure or bleeding disorders. In rats, excessively high dosages have increased total cholesterol following chronic over-consumption [122] (equivalent of × 3 the maximum daily dosage of 3 g/day n-3 recommended by the United States Food and Drug Administration in humans). Similar high-dosage n-3 (5 mL/kg/day of DHA) increased high-density lipoprotein (HDL) levels in rats after 13 weeks [180]. In humans, these lipid profile abnormalities can be detrimental to those with type 2 diabetes [192], and can lead to increased stroke risk [193], or low blood pressure [194]. For those with metabolic or heart diseases that already comorbidly present with neurodegenerative disorders (e.g. AD or Parkinson’s disease [195]), this may inadvertently increase their risk of developing a neurodegenerative disorder [190].

Finally, promoting a balanced and life-long n-3 rich diet for greater SPM bioavailability requires the interest and involvement of multiple sectors and stakeholders. The current “Western diet” contains excessive n-6 PUFAs compared to n-3 leading to an unbalanced ratio with consequences to brain health [23, 196]. A 2024 worldwide n-3 PUFA status map shows most Western countries to have low n-3 consumption including the United States, Canada, and Australia [197]. However, the National Institutes of Health (NIH) recommends daily intakes of 1.6 g for men and 1.1 g for women (respectively, ~ 3 oz/85 g and ~ 1.9 oz/55 g of Atlantic, wild, cooked salmon). The recommendation for women increases to 1.4 g during pregnancy and 1.3 g during lactation to support fetal development [198]. Promoting greater n-3 consumption for its SPM bioavailability can include promoting consumer awareness of a healthy n-3 rich diet, government campaigning, development of school programmes that encourage children to maintain healthy eating early in life and providing nutrition and dietary counselling at primary health-care facilities for the aging population [199].

## Conclusions

Our understanding of disease pathways in AD has accelerated tremendously in the last few decades, yet there is still no consensus on the underlying bioenergetic mechanisms linking lipid metabolism, chronic inflammatory microglia, and their phagocytic response in the aging brain. Advances in bioinformatics have enabled the discovery of crucial dysregulations in mitochondrial oxidative phosphorylation, neuronal apoptosis, and the microglia-mediated immune responses (e.g. *mTOR* and *TREM2*). As shown, research into early cerebral hypometabolic changes in AD exists, yet much of this assesses global or neuron-specific alterations with little interest in microglia until now. Sex-based differences in estrogen may also mediate sexual dimorphism in the risk of AD and have differential neuroprotective effects on mitochondrial respiration and microglial function, yet the role of sex in the microglial response to amyloid-β remains unclear. Nonetheless, dietary n-3-derived SPMs have been shown to support microglial energy metabolism (e.g. upregulating PPAR) and clearance of amyloid-β by facilitating a lipid mediated transition from pro-inflammatory to pro-resolving SPMs [200]. Mechanistically, this appears linked to an upregulation of *Sirt1* levels which downregulate the NF-κB inflammatory pathway signalling, thus increasing mitochondrial respiration [201]. Given these findings, interest has shifted towards the role of an n-3 enriched diet as a mechanism to ameliorate the chronic inflammation associated with AD in the growing fields of lipidomics and neurodegenerative disease.

Currently, the most widely accepted plasma and CSF biomarkers of AD include the ratio of amyloid-β_42/40_ [202], levels of p-tau_181/217_ and glial fibrillary acidic protein [203]. However, clinical use of these biomarkers is limited by their test to re-test variability, with p-tau_217_ showing the least variability [203]. Since the onset of brain mitochondrial metabolic dysfunction may begin decades before clinical symptoms, diagnostic tests can instead be strengthened by combining numerous plasma markers including plasma lipids known to be altered in AD (diacylglycerols, prostaglandins and phospholipids [204]) and mitochondria-related markers into a multi-marker panel. Translocator protein (TSPO)—a microglia-specific bioenergetic marker—plays a key role in phagocytosis, oxidative phosphorylation, ATP production, and lipid biosynthesis during neuroinflammation [205]. It is upregulated in microglia that surround amyloid-β plaques in AD brains [206], and its knockout in microglia isolated from transgenic *App* knock-in mice is associated with elevated activity of the rate-limiting glucose metabolizing enzyme, hexokinase-2 (Hk2), and impaired amyloid-β phagocytosis [205]. A multifaceted biomarker panel could include targets that are not only indicative of microgliosis (Iba1, Cd16, Mhc-II [207]) but also indicative of microglial bioenergetics (Tspo, Hk2, lactate [205]). Together with previously discussed changes in microglial phenotype and bioenergetics across disease stages, predictions can also be made on stage-specific biomarker changes. For example, in early neurodegenerative stages where microglia retain function and oxidative phosphorylation is the predominant respiratory pathway [102], we would predict blood samples will show elevated TSPO associated with more phagocytic microglia. In later stages where microglia become dysfunctional and less phagocytic [88, 93, 101], we would predict blood samples will show decreased TSPO and increased HK2, lactate, P2RY12, and MHC-II, reflecting a more hyperglycolytic, dysfunctional and pro-inflammatory state. The inclusion of mitochondria-related markers in a multifaceted biomarker panel becomes even more crucial when considering the extensive overlap of the lipidomes of patients with Parkinson’s and AD [208]. This would make mitochondrial-related markers a beneficial addition to a biomarker panel.

Developments in lipidomics provide new opportunities to assess the interactions between n-3-derived SPMs and AD, and how metabolic pathways mediate genetic risk and disease development. Further research is needed to assess the long-term benefits of n-3 derived SPMs in supporting Sirt1-mediated mitochondrial respiration in microglia and how this may impact their phagocytic response to amyloid-β in AD. Clinical studies assessing n-3 consumption show promising neuroprotective effects, reducing risk of developing AD and slowing down cognitive decline in those diagnosed. Whether these effects are largely due to the bioavailability of pro-resolving SPMs is still to be explored in humans. Nonetheless, current data from cultured microglia provide insight into how long-term bioavailability of SPMs may potentially reduce the risk of AD and illustrate the need for more experimental data using AD animal models.

## References

[1] Brown L, J Li, HA La. The economic and societal cost of Alzheimer's Disease in Australia 2021-2041. Canberra: University of Canberr; 2022.

[2] Alzheimer's disease facts and figures. Alzheimers Dement. 2020;16(3):385–580.

[3] Prince M, et al. The global prevalence of dementia: a systematic review and metaanalysis. Alzheimers Dement. 2013;9(1):63-75.e2. 23305823 10.1016/j.jalz.2012.11.007

[4] Wimo A, et al. The worldwide economic impact of dementia 2010. Alzheimers Dement. 2013;9(1):1-11.e3. 23305821 10.1016/j.jalz.2012.11.006

[5] Neddens J, et al. Phosphorylation of different tau sites during progression of Alzheimer’s disease. Acta Neuropathol Commun. 2018;6(1):52. 29958544 10.1186/s40478-018-0557-6 PMC6027763

[6] Terni B, et al. Mitochondrial ATP-synthase in the entorhinal cortex is a target of oxidative stress at stages I/II of Alzheimer’s disease pathology. Brain Pathol. 2010;20(1):222–33. 19298596 10.1111/j.1750-3639.2009.00266.x PMC8094794

[7] Manczak M, et al. Differential expression of oxidative phosphorylation genes in patients with Alzheimer’s disease: implications for early mitochondrial dysfunction and oxidative damage. Neuromolecular Med. 2004;5(2):147–62. 15075441 10.1385/NMM:5:2:147

[8] Vaillant-Beuchot L, et al. Accumulation of amyloid precursor protein C-terminal fragments triggers mitochondrial structure, function, and mitophagy defects in Alzheimer’s disease models and human brains. Acta Neuropathol. 2021;141(1):39–65. 33079262 10.1007/s00401-020-02234-7 PMC7785558

[9] Du F, et al. PINK1 signalling rescues amyloid pathology and mitochondrial dysfunction in Alzheimer’s disease. Brain. 2017;140(12):3233–51. 29077793 10.1093/brain/awx258 PMC5841141

[10] Armand-Ugon M, et al. Reduced Mitochondrial Activity is Early and Steady in the Entorhinal Cortex but it is Mainly Unmodified in the Frontal Cortex in Alzheimer’s Disease. Curr Alzheimer Res. 2017;14(12):1327–34. 28474567 10.2174/1567205014666170505095921

[11] Pinho CM, Teixeira PF, Glaser E. Mitochondrial import and degradation of amyloid-β peptide. Biochimica et Biophysica Acta (BBA) -. Bioenergetics. 2014;1837(7):1069–74. 10.1016/j.bbabio.2014.02.007 24561226

[12] Du H, et al. Cyclophilin D deficiency attenuates mitochondrial and neuronal perturbation and ameliorates learning and memory in Alzheimer’s disease. Nat Med. 2008;14(10):1097–105. 18806802 10.1038/nm.1868 PMC2789841

[13] Hauptmann S, et al. Mitochondrial dysfunction: An early event in Alzheimer pathology accumulates with age in AD transgenic mice. Neurobiol Aging. 2009;30(10):1574–86. 18295378 10.1016/j.neurobiolaging.2007.12.005

[14] Yasumoto T, et al. High molecular weight amyloid β1-42 oligomers induce neurotoxicity via plasma membrane damage. FASEB J. 2019;33(8):9220–34. 31084283 10.1096/fj.201900604R

[15] Rosen KM, et al. Parkin reverses intracellular β-amyloid accumulation and its negative effects on proteasome function. J Neurosci Res. 2010;88(1):167–78. 19610108 10.1002/jnr.22178 PMC2844439

[16] Caspersen C, et al. Mitochondrial Aβ: a potential focal point for neuronal metabolic dysfunction in Alzheimer’s disease. FASEB J. 2005;19(14):2040–1. 16210396 10.1096/fj.05-3735fje

[17] Chen Z, Zhong C. Decoding Alzheimer’s disease from perturbed cerebral glucose metabolism: implications for diagnostic and therapeutic strategies. Prog Neurobiol. 2013;108:21–43. 23850509 10.1016/j.pneurobio.2013.06.004

[18] Paranjpe MD, et al. The effect of ApoE ε4 on longitudinal brain region-specific glucose metabolism in patients with mild cognitive impairment: a FDG-PET study. Neuroimage Clin. 2019;22: 101795. 30991617 10.1016/j.nicl.2019.101795 PMC6449776

[19] Reiman EM, et al. Functional brain abnormalities in young adults at genetic risk for late-onset Alzheimer’s dementia. Proc Natl Acad Sci U S A. 2004;101(1):284–9. 14688411 10.1073/pnas.2635903100 PMC314177

[20] Weise CM, et al. Left lateralized cerebral glucose metabolism declines in amyloid-β positive persons with mild cognitive impairment. Neuroimage Clin. 2018;20:286–96. 30101060 10.1016/j.nicl.2018.07.016 PMC6084012

[21] Loving BA, Bruce KD. Lipid and Lipoprotein Metabolism in Microglia. Front Physiol. 2020;11: 393. 32411016 10.3389/fphys.2020.00393 PMC7198855

[22] DiNicolantonio JJ, O'Keefe JH, The Importance of Marine Omega-3s for Brain Development and the Prevention and Treatment of Behavior, Mood, and Other Brain Disorders. Nutrients. 2020;12(8). 10.3390/nu12082333 PMC7468918 32759851

[23] Spencer SJ, et al. Food for thought: how nutrition impacts cognition and emotion. NPJ Science of Food. 2017;1(1):7. 31304249 10.1038/s41538-017-0008-y PMC6550267

[24] Serhan CN. Pro-resolving lipid mediators are leads for resolution physiology. Nature. 2014;510(7503):92–101. 24899309 10.1038/nature13479 PMC4263681

[25] Ponce J, et al. Role of Specialized Pro-resolving Mediators in Reducing Neuroinflammation in Neurodegenerative Disorders. Frontiers in Aging Neuroscience. 2022;14:780811. 35250536 10.3389/fnagi.2022.780811 PMC8891627

[26] Ciccarelli R, et al. Cysteinyl-leukotrienes are released from astrocytes and increase astrocyte proliferation and glial fibrillary acidic protein via cys-LT1 receptors and mitogen-activated protein kinase pathway. Eur J Neurosci. 2004;20(6):1514–24. 15355318 10.1111/j.1460-9568.2004.03613.x

[27] Yu SY, et al. Cysteinyl leukotriene receptor 1 mediates LTD4-induced activation of mouse microglial cells in vitro. Acta Pharmacol Sin. 2014;35(1):33–40. 24141567 10.1038/aps.2013.130 PMC4075749

[28] Li C, et al. Role of resolvins in the inflammatory resolution of neurological diseases. Front Pharmacol. 2020;11:612. 32457616 10.3389/fphar.2020.00612 PMC7225325

[29] Chiurchiù V, et al. Proresolving lipid mediators resolvin D1, resolvin D2, and maresin 1 are critical in modulating T cell responses. Science Translational Medicine. 2016;8(353):353ra111-353ra111. 10.1126/scitranslmed.aaf7483 PMC5149396 27559094

[30] Dalli J, Serhan CN. Specific lipid mediator signatures of human phagocytes: microparticles stimulate macrophage efferocytosis and pro-resolving mediators. Blood. 2012;120(15):e60–72. 22904297 10.1182/blood-2012-04-423525 PMC3471524

[31] Shang P, et al. Inflammation resolution and specialized pro-resolving lipid mediators in CNS diseases. Expert Opin Ther Targets. 2019;23(11):967–86. 31711309 10.1080/14728222.2019.1691525

[32] Wu Y, et al. Aspirin-triggered lipoxin A₄ attenuates lipopolysaccharide-induced intracellular ROS in BV2 microglia cells by inhibiting the function of NADPH oxidase. Neurochem Res. 2012;37(8):1690–6. 22552474 10.1007/s11064-012-0776-3

[33] Decker Y, McBean G, Godson C. Lipoxin A4 inhibits IL-1beta-induced IL-8 and ICAM-1 expression in 1321N1 human astrocytoma cells. Am J Physiol Cell Physiol. 2009;296(6):C1420–7. 19357230 10.1152/ajpcell.00380.2008

[34] Wu J, et al. Lipoxin A4 inhibits the production of proinflammatory cytokines induced by β-amyloid in vitro and in vivo. Biochem Biophys Res Commun. 2011;408(3):382–7. 21501589 10.1016/j.bbrc.2011.04.013

[35] Zhu M, et al. Pro-Resolving Lipid Mediators Improve Neuronal Survival and Increase Aβ42 Phagocytosis. Mol Neurobiol. 2016;53(4):2733–49. 26650044 10.1007/s12035-015-9544-0 PMC4824659

[36] Mizwicki MT, et al. 1α,25-dihydroxyvitamin D3 and resolvin D1 retune the balance between amyloid-β phagocytosis and inflammation in Alzheimer’s disease patients. J Alzheimers Dis. 2013;34(1):155–70. 23186989 10.3233/JAD-121735 PMC4040018

[37] Zhao Y, et al. Docosahexaenoic acid-derived neuroprotectin D1 induces neuronal survival via secretase- and PPARγ-mediated mechanisms in Alzheimer’s disease models. PLoS ONE. 2011;6(1): e15816. 21246057 10.1371/journal.pone.0015816 PMC3016440

[38] Yin P, et al. Maresin 1 Improves Cognitive Decline and Ameliorates Inflammation in a Mouse Model of Alzheimer’s Disease. Front Cell Neurosci. 2019;13. 10.3389/fncel.2019.00466 PMC6803487 31680874

[39] Perrot CY, et al. Prostaglandin E2 breaks down pericyte-endothelial cell interaction via EP1 and EP4-dependent downregulation of pericyte N-cadherin, connexin-43, and R-Ras. Sci Rep. 2020;10(1):11186. 32636414 10.1038/s41598-020-68019-w PMC7341885

[40] Quan Y, Jiang J, Dingledine R. EP2 receptor signaling pathways regulate classical activation of microglia. J Biol Chem. 2013;288(13):9293–302. 23404506 10.1074/jbc.M113.455816 PMC3611000

[41] Kunori S, et al. A novel role of prostaglandin E2 in neuropathic pain: blockade of microglial migration in the spinal cord. Glia. 2011;59(2):208–18. 21125641 10.1002/glia.21090

[42] Liang X, et al. The prostaglandin E2 EP2 receptor accelerates disease progression and inflammation in a model of amyotrophic lateral sclerosis. Ann Neurol. 2008;64(3):304–14. 18825663 10.1002/ana.21437 PMC2766522

[43] Yan A, et al. Thromboxane A2 receptor antagonist SQ29548 reduces ischemic stroke-induced microglia/macrophages activation and enrichment, and ameliorates brain injury. Sci Rep. 2016;6(1):35885. 27775054 10.1038/srep35885 PMC5075919

[44] Zhao Z, et al. Hyperglycemia via activation of thromboxane A2 receptor impairs the integrity and function of blood-brain barrier in microvascular endothelial cells. Oncotarget. 2017;8(18):30030–8. 28415790 10.18632/oncotarget.16273 PMC5444723

[45] Barone FC, et al. Time-related changes in myeloperoxidase activity and leukotriene B4 receptor binding reflect leukocyte influx in cerebral focal stroke. Mol Chem Neuropathol. 1995;24(1):13–30. 7755844 10.1007/BF03160109

[46] Black KL, Hoff JT. Leukotrienes increase blood-brain barrier permeability following intraparenchymal injections in rats. Ann Neurol. 1985;18(3):349–51. 2996417 10.1002/ana.410180313

[47] Krashia P, et al. Blunting neuroinflammation with resolvin D1 prevents early pathology in a rat model of Parkinson’s disease. Nat Commun. 2019;10(1):3945. 31477726 10.1038/s41467-019-11928-w PMC6718379

[48] Xu J, et al. Resolvin D1 Attenuates Mpp+-Induced Parkinson Disease via Inhibiting Inflammation in PC12 Cells. Med Sci Monit. 2017;23:2684–91. 28572562 10.12659/MSM.901995 PMC5465971

[49] Fonteh AN, et al. Polyunsaturated Fatty Acid Composition of Cerebrospinal Fluid Fractions Shows Their Contribution to Cognitive Resilience of a Pre-symptomatic Alzheimer’s Disease Cohort. Frontiers in Physiology. 2020;11:83. 32116789 10.3389/fphys.2020.00083 PMC7034243

[50] Fiala M, et al. ω-3 Supplementation increases amyloid-β phagocytosis and resolvin D1 in patients with minor cognitive impairment. FASEB J. 2015;29(7):2681–9. 25805829 10.1096/fj.14-264218

[51] Kantarci A, et al. Combined administration of resolvin E1 and lipoxin A4 resolves inflammation in a murine model of Alzheimer’s disease. Exp Neurol. 2018;300:111–20. 29126887 10.1016/j.expneurol.2017.11.005

[52] Wang L, et al. Resolvin D1 attenuates sepsis induced acute kidney injury targeting mitochondria and NF-κB signaling pathway. Heliyon. 2022;8(12): e12269. 36578378 10.1016/j.heliyon.2022.e12269 PMC9791840

[53] Calderin EP, et al. Exercise-induced specialized proresolving mediators stimulate AMPK phosphorylation to promote mitochondrial respiration in macrophages. Mol Metab. 2022;66: 101637. 36400404 10.1016/j.molmet.2022.101637 PMC9719872

[54] Xue B, et al. Omega-3 Polyunsaturated Fatty Acids Antagonize Macrophage Inflammation via Activation of AMPK/SIRT1 Pathway. PLoS ONE. 2012;7(10): e45990. 23071533 10.1371/journal.pone.0045990 PMC3465287

[55] Yang Y, et al. Maresin conjugates in tissue regeneration 1 prevents lipopolysaccharide-induced cardiac dysfunction through improvement of mitochondrial biogenesis and function. Biochem Pharmacol. 2020;177: 114005. 32360364 10.1016/j.bcp.2020.114005

[56] Inomata R, et al. Resolvin D4 mitigates lipopolysaccharide-induced lung injury in mice. Prostaglandins Leukot Essent Fatty Acids. 2024;203: 102652. 39368237 10.1016/j.plefa.2024.102652

[57] Inoue T, et al. Omega-3 polyunsaturated fatty acids suppress the inflammatory responses of lipopolysaccharide-stimulated mouse microglia by activating SIRT1 pathways. Biochim Biophys Acta Mol Cell Biol Lipids. 2017;1862(5):552–60. 28254441 10.1016/j.bbalip.2017.02.010

[58] Jiang S, et al. LXA4 attenuates perioperative neurocognitive disorders by suppressing neuroinflammation and oxidative stress. Int Immunopharmacol. 2023;123: 110788. 37591120 10.1016/j.intimp.2023.110788

[59] Jung TW, et al. Maresin 1 attenuates NAFLD by suppression of endoplasmic reticulum stress via AMPK-SERCA2b pathway. J Biol Chem. 2018;293(11):3981–8. 29414781 10.1074/jbc.RA117.000885 PMC5857988

[60] Lee D. Sirt1 as a New Therapeutic Target in Metabolic and Age-Related Diseases. Chonnam Medical Journal. 2010;46:46.

[61] Nemeth Z, Kiss E, Takacs I. The Role of Epigenetic Regulator SIRT1 in Balancing the Homeostasis and Preventing the Formation of Specific “Soil” of Metabolic Disorders and Related Cancers. FBL. 2022;27:27(9). 36224002 10.31083/j.fbl2709253

[62] Ohira T, et al. Resolvin E1 receptor activation signals phosphorylation and phagocytosis. J Biol Chem. 2010;285(5):3451–61. 19906641 10.1074/jbc.M109.044131 PMC2823415

[63] Wan X, Garg NJ. Sirtuin control of mitochondrial dysfunction, oxidative stress, and inflammation in chagas disease models. Front Cell Infect Microbiol. 2021;11:693051. 34178728 10.3389/fcimb.2021.693051 PMC8221535

[64] Wickstead ES, et al. Stimulation of the pro-resolving receptor Fpr2 reverses inflammatory microglial activity by suppressing NFκB activity. Int J Mol Sci. 2023;24(21):15996. 37958978 10.3390/ijms242115996 PMC10649357

[65] Wu Q-J, et al. The sirtuin family in health and disease. Signal Transduct Target Ther. 2022;7(1):402. 36581622 10.1038/s41392-022-01257-8 PMC9797940

[66] Xu X, et al. Annexin A1 protects against cerebral ischemia–reperfusion injury by modulating microglia/macrophage polarization via FPR2/ALX-dependent AMPK-mTOR pathway. J Neuroinflammation. 2021;18(1):119. 34022892 10.1186/s12974-021-02174-3 PMC8140477

[67] Zhao M, et al. Maresin-1 and its receptors RORα/LGR6 as potential therapeutic target for respiratory diseases. Pharmacol Res. 2022;182: 106337. 35781060 10.1016/j.phrs.2022.106337

[68] Kerr JS, et al. Mitophagy and Alzheimer’s Disease: Cellular and Molecular Mechanisms. Trends Neurosci. 2017;40(3):151–66. 28190529 10.1016/j.tins.2017.01.002 PMC5341618

[69] Hong S, et al. Complement and microglia mediate early synapse loss in Alzheimer mouse models. Science. 2016;352(6286):712–6. 27033548 10.1126/science.aad8373 PMC5094372

[70] Backes H, et al. Glucose consumption of inflammatory cells masks metabolic deficits in the brain. Neuroimage. 2016;128:54–62. 26747749 10.1016/j.neuroimage.2015.12.044 PMC4767221

[71] Mary A, et al. Mitophagy in Alzheimer’s disease: Molecular defects and therapeutic approaches. Mol Psychiatry. 2023;28(1):202–16. 35665766 10.1038/s41380-022-01631-6 PMC9812780

[72] Hyder F, Rothman DL, Bennett MR. Cortical energy demands of signaling and nonsignaling components in brain are conserved across mammalian species and activity levels. Proc Natl Acad Sci. 2013;110(9):3549–54. 23319606 10.1073/pnas.1214912110 PMC3587194

[73] Martín-Maestro P, et al. PARK2 enhancement is able to compensate mitophagy alterations found in sporadic Alzheimer’s disease. Hum Mol Genet. 2015;25(4):792–806. 26721933 10.1093/hmg/ddv616 PMC4743695

[74] Pappatà S, Salvatore E, Postiglione A. In Vivo Imaging of Neurotransmission and Brain Receptors in Dementia. J Neuroimaging. 2008;18(2):111–24. 18380693 10.1111/j.1552-6569.2007.00194.x

[75] Wang W, et al. Mitochondria dysfunction in the pathogenesis of Alzheimer’s disease: recent advances. Mol Neurodegener. 2020;15(1):30. 32471464 10.1186/s13024-020-00376-6 PMC7257174

[76] Liang WS, et al. Alzheimer’s disease is associated with reduced expression of energy metabolism genes in posterior cingulate neurons. Proc Natl Acad Sci U S A. 2008;105(11):4441–6. 18332434 10.1073/pnas.0709259105 PMC2393743

[77] Madore C, et al. Essential omega-3 fatty acids tune microglial phagocytosis of synaptic elements in the mouse developing brain. Nat Commun. 2020;11(1):6133. 33257673 10.1038/s41467-020-19861-z PMC7704669

[78] Engl E, Attwell D. Non-signalling energy use in the brain. J Physiol. 2015;593(16):3417–29. 25639777 10.1113/jphysiol.2014.282517 PMC4560575

[79] Nair S, et al. Lipopolysaccharide-induced alteration of mitochondrial morphology induces a metabolic shift in microglia modulating the inflammatory response in vitro and in vivo. Glia. 2019;67(6):1047–61. 30637805 10.1002/glia.23587

[80] Puleston DJ, Villa M, Pearce EL. Ancillary Activity: Beyond Core Metabolism in Immune Cells. Cell Metab. 2017;26(1):131–41. 28683280 10.1016/j.cmet.2017.06.019 PMC5546226

[81] Vats D, et al. Oxidative metabolism and PGC-1β attenuate macrophage-mediated inflammation. Cell Metab. 2006;4(1):13–24. 16814729 10.1016/j.cmet.2006.05.011 PMC1904486

[82] Gimeno-Bayón J, et al. Glucose pathways adaptation supports acquisition of activated microglia phenotype. J Neurosci Res. 2014;92(6):723–31. 24510633 10.1002/jnr.23356

[83] Tan Z, et al. Pyruvate dehydrogenase kinase 1 participates in macrophage polarization via regulating glucose metabolism. J Immunol. 2015;194(12):6082–9. 25964487 10.4049/jimmunol.1402469 PMC4458459

[84] Baik SH, et al. A Breakdown in Metabolic Reprogramming Causes Microglia Dysfunction in Alzheimer’s Disease. Cell Metab. 2019;30(3):493-507.e6. 31257151 10.1016/j.cmet.2019.06.005

[85] Ulland TK, et al. TREM2 Maintains Microglial Metabolic Fitness in Alzheimer’s Disease. Cell. 2017;170(4):649-663.e13. 28802038 10.1016/j.cell.2017.07.023 PMC5573224

[86] Galluzzi L, et al. Metabolic control of autophagy. Cell. 2014;159(6):1263–76. 25480292 10.1016/j.cell.2014.11.006 PMC4500936

[87] Saxton RA, Sabatini DM. mTOR Signaling in Growth, Metabolism, and Disease. Cell. 2017;168(6):960–76. 28283069 10.1016/j.cell.2017.02.004 PMC5394987

[88] Hickman S, et al. Microglia in neurodegeneration. Nat Neurosci. 2018;21(10):1359–69. 30258234 10.1038/s41593-018-0242-x PMC6817969

[89] Condello C, et al. Microglia constitute a barrier that prevents neurotoxic protofibrillar Aβ42 hotspots around plaques. Nat Commun. 2015;6:6176. 25630253 10.1038/ncomms7176 PMC4311408

[90] Freemerman AJ, et al. Metabolic reprogramming of macrophages: glucose transporter 1 (GLUT1)-mediated glucose metabolism drives a proinflammatory phenotype. J Biol Chem. 2014;289(11):7884–96. 24492615 10.1074/jbc.M113.522037 PMC3953299

[91] Karki R, Kodamullil AT, Hofmann-Apitius M. Comorbidity Analysis between Alzheimer’s Disease and Type 2 Diabetes Mellitus (T2DM) Based on Shared Pathways and the Role of T2DM Drugs. J Alzheimers Dis. 2017;60(2):721–31. 28922161 10.3233/JAD-170440 PMC5611890

[92] Cianciulli A, et al. Microglia mediated neuroinflammation: focus on PI3K modulation. Biomolecules. 2020;276(1):137. 10.3390/biom10010137 PMC7022557 31947676

[93] Brooks WM, et al. Gene expression profiles of metabolic enzyme transcripts in Alzheimer’s disease. Brain Res. 2007;1127(1):127–35. 17109828 10.1016/j.brainres.2006.09.106

[94] Liang H, Ward WF. PGC-1alpha: a key regulator of energy metabolism. Adv Physiol Educ. 2006;30(4):145–51. 17108241 10.1152/advan.00052.2006

[95] Koenig S, et al. Leptin is involved in age-dependent changes in response to systemic inflammation in the rat. Brain Behav Immun. 2014;36:128–38. 24513873 10.1016/j.bbi.2013.10.019

[96] Qin W, et al. PGC-1alpha expression decreases in the Alzheimer disease brain as a function of dementia. Arch Neurol. 2009;66(3):352–61. 19273754 10.1001/archneurol.2008.588 PMC3052997

[97] Fang EF, et al. Mitophagy inhibits amyloid-β and tau pathology and reverses cognitive deficits in models of Alzheimer’s disease. Nat Neurosci. 2019;22(3):401–12. 30742114 10.1038/s41593-018-0332-9 PMC6693625

[98] Sorrentino V, et al. Enhancing mitochondrial proteostasis reduces amyloid-β proteotoxicity. Nature. 2017;552(7684):187–93. 29211722 10.1038/nature25143 PMC5730497

[99] Zhang L, et al. Potential hippocampal genes and pathways involved in Alzheimer’s disease: a bioinformatic analysis. Genet Mol Res. 2015;14(2):7218–32. 26125932 10.4238/2015.June.29.15

[100] Miller B, et al. Mitochondrial DNA variation in Alzheimer’s disease reveals a unique microprotein called SHMOOSE. Mol Psychiatry. 2023;28(4):1813–26. 36127429 10.1038/s41380-022-01769-3 PMC10027624

[101] Minjarez B, et al. Identification of proteins that are differentially expressed in brains with Alzheimer’s disease using iTRAQ labeling and tandem mass spectrometry. J Proteomics. 2016;139:103–21. 27012543 10.1016/j.jprot.2016.03.022

[102] Mastroeni D, et al. Nuclear but not mitochondrial-encoded oxidative phosphorylation genes are altered in aging, mild cognitive impairment, and Alzheimer’s disease. Alzheimers Dement. 2017;13(5):510–9. 27793643 10.1016/j.jalz.2016.09.003 PMC5967608

[103] Bubber P, et al. Mitochondrial abnormalities in Alzheimer brain: mechanistic implications. Ann Neurol. 2005;57(5):695–703. 15852400 10.1002/ana.20474

[104] Martínez RAS, et al. GTP energy dependence of endocytosis and autophagy in the aging brain and Alzheimer’s disease. Geroscience. 2023;45(2):757–80. 36622562 10.1007/s11357-022-00717-x PMC9886713

[105] Burnstock G. Purinergic Signalling—an overview, in purinergic signalling in neuron–glia interactions. 2006:26-53.

[106] Mei SY, et al. Microglial purinergic signaling in Alzheimer’s disease. Purinergic Signalling. 2024. 10.1007/s11302-024-10029-8 PMC12454253 38910192

[107] Albasanz JL, et al. Up-regulation of adenosine receptors in the frontal cortex in Alzheimer’s disease. Brain Pathol. 2008;18(2):211–9. 18241242 10.1111/j.1750-3639.2007.00112.x PMC8095610

[108] Alonso-Andrés P, et al. Purine-related metabolites and their converting enzymes are altered in frontal, parietal and temporal cortex at early stages of Alzheimer’s disease pathology. Brain Pathol. 2018;28(6):933–46. 29363833 10.1111/bpa.12592 PMC8028663

[109] Adav SS, Park JE, Sze SK. Quantitative profiling brain proteomes revealed mitochondrial dysfunction in Alzheimer’s disease. Mol Brain. 2019;12(1):8. 30691479 10.1186/s13041-019-0430-y PMC6350377

[110] Lopes CR, et al. Downregulation of Sirtuin 1 Does Not Account for the Impaired Long-Term Potentiation in the Prefrontal Cortex of Female APPswe/PS1dE9 Mice Modelling Alzheimer's Disease. Int J Mol Sci. 2023;24(8). 10.3390/ijms24086968 PMC10139121 37108131

[111] Li B, et al. Impact of neural stem cell-derived extracellular vesicles on mitochondrial dysfunction, sirtuin 1 level, and synaptic deficits in Alzheimer’s disease. J Neurochem. 2020;154(5): e15001. 10.1111/jnc.15001 32145065

[112] Hadar A, et al. SIRT1, miR-132 and miR-212 link human longevity to Alzheimer’s Disease. Sci Rep. 2018;8(1):8465. 29855513 10.1038/s41598-018-26547-6 PMC5981646

[113] Silva DF, et al. Mitochondrial Metabolism Power SIRT2-Dependent Deficient Traffic Causing Alzheimer’s-Disease Related Pathology. Mol Neurobiol. 2017;54(6):4021–40. 27311773 10.1007/s12035-016-9951-x

[114] Bai N, et al. Inhibition of SIRT2 promotes APP acetylation and ameliorates cognitive impairment in APP/PS1 transgenic mice. Cell Rep. 2022;40(2): 111062. 35830807 10.1016/j.celrep.2022.111062

[115] Wongchitrat P, et al. Alterations in the expression of amyloid precursor protein cleaving enzymes mRNA in Alzheimer peripheral blood. Curr Alzheimer Res. 2019;16(1):29–38. 30411686 10.2174/1567205015666181109103742

[116] Shi H-Z, et al. The potential benefits of PGC-1α in treating Alzheimer’s disease are dependent on the integrity of the LLKYL L3 motif: Effect of regulating mitochondrial axonal transportation. Exp Gerontol. 2024;194: 112514. 38971132 10.1016/j.exger.2024.112514

[117] Wang J, et al. PGC-1α reduces Amyloid-β deposition in Alzheimer’s disease: effect of increased VDR expression. Neurosci Lett. 2021;744:135598. 33373677 10.1016/j.neulet.2020.135598

[118] Katsouri L, et al. PPARγ Co-Activator-1α (PGC-1α) Reduces Amyloid-β Generation Through a PPARγ-Dependent Mechanism. Journal of Alzheimer’s Disease. 2011;25(1):151–62. 21358044 10.3233/JAD-2011-101356

[119] Francis BM, et al. Reduced levels of mitochondrial complex I subunit NDUFB8 and linked complex I + III oxidoreductase activity in the TgCRND8 mouse model of Alzheimer’s disease. J Alzheimers Dis. 2014;39(2):347–55. 24217272 10.3233/JAD-131499

[120] Gao H, et al. Ndufs4 knockout induces transcriptomic signatures of Alzheimer’s Diseases that are partially reversed by mitochondrial complex I inhibitor. bioRxiv, 2024: p. 2024.02.20.581247.

[121] Xie C, et al. Amelioration of Alzheimer’s disease pathology by mitophagy inducers identified via machine learning and a cross-species workflow. Nature Biomedical Engineering. 2022;6(1):76–93. 34992270 10.1038/s41551-021-00819-5 PMC8782726

[122] Lunnon K, et al. Mitochondrial genes are altered in blood early in Alzheimer’s disease. Neurobiol Aging. 2017;53:36–47. 28208064 10.1016/j.neurobiolaging.2016.12.029

[123] Rudisch DM, et al. Early ultrasonic vocalization deficits and related thyroarytenoid muscle pathology in the transgenic TgF344-AD rat model of Alzheimer’s disease. Front Behav Neurosci. 2024;17:1294648. 38322496 10.3389/fnbeh.2023.1294648 PMC10844490

[124] Bi R, et al. Genetic association of the cytochrome c oxidase-related genes with Alzheimer’s disease in Han Chinese. Neuropsychopharmacology. 2018;43(11):2264–76. 30054583 10.1038/s41386-018-0144-3 PMC6135758

[125] Morello G, et al. Transcriptomic Analysis in the Hippocampus and Retina of Tg2576 AD Mice Reveals Defective Mitochondrial Oxidative Phosphorylation and Recovery by Tau 12A12mAb Treatment. Cells. 2023;12(18):2254. 37759477 10.3390/cells12182254 PMC10527038

[126] Martín-Maestro P, et al. Autophagy Induction by Bexarotene Promotes Mitophagy in Presenilin 1 Familial Alzheimer’s Disease iPSC-Derived Neural Stem Cells. Mol Neurobiol. 2019;56(12):8220–36. 31203573 10.1007/s12035-019-01665-y PMC6842097

[127] Orr AL, et al. Neuronal Apolipoprotein E4 Expression Results in Proteome-Wide Alterations and Compromises Bioenergetic Capacity by Disrupting Mitochondrial Function. J Alzheimers Dis. 2019;68(3):991–1011. 30883359 10.3233/JAD-181184 PMC6481541

[128] González-Domínguez R, et al. Region-specific metabolic alterations in the brain of the APP/PS1 transgenic mice of Alzheimer’s disease. Biochim Biophysic Acta (BBA) - Mol Basis Dis. 2014;1842(12, Part A):2395–402. 10.1016/j.bbadis.2014.09.014 25281826

[129] Saito ER, et al. Alzheimer’s disease alters oligodendrocytic glycolytic and ketolytic gene expression. Alzheimers Dement. 2021;17(9):1474–86. 33650792 10.1002/alz.12310 PMC8410881

[130] Qiu Z, et al. The significance of glycolysis index and its correlations with immune infiltrates in Alzheimer’s disease. Frontiers in Immunology. 2022;13:960906. 36353631 10.3389/fimmu.2022.960906 PMC9637950

[131] Zhang M, et al. Lactate Deficit in an Alzheimer Disease Mouse Model: The Relationship With Neuronal Damage. J Neuropathol Exp Neurol. 2018;77(12):1163–76. 30383244 10.1093/jnen/nly102

[132] Niccoli T, et al. Activating transcription factor 4-dependent lactate dehydrogenase activation as a protective response to amyloid beta toxicity. Brain Commun. 2021;3(2):fcab053. 33977265 10.1093/braincomms/fcab053 PMC8093921

[133] Shaerzadeh F, Motamedi F, Khodagholi F. Inhibition of akt phosphorylation diminishes mitochondrial biogenesis regulators, tricarboxylic acid cycle activity and exacerbates recognition memory deficit in rat model of Alzheimer’s disease. Cell Mol Neurobiol. 2014;34(8):1223–33. 25135709 10.1007/s10571-014-0099-9 PMC11488940

[134] Correas AG, et al. Glucose 6 phosphate dehydrogenase overexpression rescues the loss of cognition in the double transgenic APP/PS1 mouse model of Alzheimer’s disease. Redox Biol. 2024;75: 103242. 38908073 10.1016/j.redox.2024.103242 PMC11253689

[135] Jia D, Wang F, Yu H. Systemic alterations of tricarboxylic acid cycle enzymes in Alzheimer’s disease. Front Neurosci. 2023;17:1206688. 37575300 10.3389/fnins.2023.1206688 PMC10413568

[136] Park YH, et al. Omega-3 Fatty Acid-Type Docosahexaenoic Acid Protects against Aβ-Mediated Mitochondrial Deficits and Pathomechanisms in Alzheimer’s Disease-Related Animal Model. Int J Mol Sci. 2020;21(11): 3879. 32486013 10.3390/ijms21113879 PMC7312360

[137] Kosgei VJ, et al. Sirt1-PPARS cross-talk in complex metabolic diseases and inherited disorders of the one carbon metabolism. Cells. 2020;9(8):1882. 32796716 10.3390/cells9081882 PMC7465293

[138] Theendakara V, et al. Neuroprotective Sirtuin ratio reversed by ApoE4. Proc Natl Acad Sci U S A. 2013;110(45):18303–8. 24145446 10.1073/pnas.1314145110 PMC3831497

[139] Julien C, et al. Sirtuin 1 reduction parallels the accumulation of tau in Alzheimer disease. J Neuropathol Exp Neurol. 2009;68(1):48–58. 19104446 10.1097/NEN.0b013e3181922348 PMC2813570

[140] Koo J-H, et al. Treadmill exercise decreases amyloid-β burden possibly via activation of SIRT-1 signaling in a mouse model of Alzheimer’s disease. Exp Neurol. 2017;288:142–52. 27889467 10.1016/j.expneurol.2016.11.014

[141] Zhao N, Xia J, Xu B. Physical exercise may exert its therapeutic influence on Alzheimer’s disease through the reversal of mitochondrial dysfunction via SIRT1-FOXO1/3-PINK1-Parkin-mediated mitophagy. J Sport Health Sci. 2021;10(1):1–3. 32861777 10.1016/j.jshs.2020.08.009 PMC7856556

[142] Majeed Y, et al. SIRT1 promotes lipid metabolism and mitochondrial biogenesis in adipocytes and coordinates adipogenesis by targeting key enzymatic pathways. Sci Rep. 2021;11(1):8177. 33854178 10.1038/s41598-021-87759-x PMC8046990

[143] Guo P, et al. Deciphering and engineering the polyunsaturated fatty acid synthase pathway from eukaryotic microorganisms. Front Bioeng Biotechnol. 2022;10: 1052785. 36452206 10.3389/fbioe.2022.1052785 PMC9702510

[144] Gillum MP, et al. SirT1 regulates adipose tissue inflammation. Diabetes. 2011;60(12):3235–45. 22110092 10.2337/db11-0616 PMC3219953

[145] Kumar R, et al. Sirtuin1: A Promising Serum Protein Marker for Early Detection of Alzheimer’s Disease. PLoS ONE. 2013;8(4): e61560. 23613875 10.1371/journal.pone.0061560 PMC3628714

[146] Campagna J, et al. A small molecule ApoE4-targeted therapeutic candidate that normalizes sirtuin 1 levels and improves cognition in an Alzheimer’s disease mouse model. Sci Rep. 2018;8(1):17574. 30514854 10.1038/s41598-018-35687-8 PMC6279743

[147] Xue B, Kahn BB. AMPK integrates nutrient and hormonal signals to regulate food intake and energy balance through effects in the hypothalamus and peripheral tissues. J Physiol. 2006;574(Pt 1):73–83. 16709629 10.1113/jphysiol.2006.113217 PMC1817809

[148] Karabatsiakis A, et al. Comparative Analysis of Temperature Effects on Mitochondrial Bioenergetics in Peripheral Blood Mononuclear Cells of Individuals with and without Depression. 2023.

[149] Park YH, et al. Omega-3 Fatty Acid-Type Docosahexaenoic Acid Protects against Aβ-Mediated Mitochondrial Deficits and Pathomechanisms in Alzheimer’s Disease-Related Animal Model. Int J Mol Sci. 2020;21(11):3879. 32486013 10.3390/ijms21113879 PMC7312360

[150] Schebb NH, et al. Formation, Signaling and Occurrence of Specialized Pro-Resolving Lipid Mediators-What is the Evidence so far? Front Pharmacol. 2022;13: 838782. 35308198 10.3389/fphar.2022.838782 PMC8924552

[151] Seshadri S, et al. Lifetime risk of dementia and Alzheimer’s disease. The impact of mortality on risk estimates in the Framingham Study. Neurology. 1997;49(6):1498–504. 9409336 10.1212/wnl.49.6.1498

[152] Goyal MS, et al. Persistent metabolic youth in the aging female brain. Proc Natl Acad Sci. 2019;116(8):3251–5. 30718410 10.1073/pnas.1815917116 PMC6386682

[153] Silaidos C, et al. Sex-associated differences in mitochondrial function in human peripheral blood mononuclear cells (PBMCs) and brain. Biol Sex Differ. 2018;9(1):34. 30045765 10.1186/s13293-018-0193-7 PMC6060503

[154] Ide T, et al. Greater oxidative stress in healthy young men compared with premenopausal women. Arterioscler Thromb Vasc Biol. 2002;22(3):438–42. 11884287 10.1161/hq0302.104515

[155] Irwin RW, et al. Selective Oestrogen Receptor Modulators Differentially Potentiate Brain Mitochondrial Function. J Neuroendocrinol. 2012;24(1):236–48. 22070562 10.1111/j.1365-2826.2011.02251.x PMC3264398

[156] Ventura-Clapier R, et al. Mitochondria: a central target for sex differences in pathologies. Clin Sci. 2017;131(9):803–22. 10.1042/CS20160485 28424375

[157] Jamwal S, Blackburn JK, Elsworth JD. PPARγ/PGC1α signaling as a potential therapeutic target for mitochondrial biogenesis in neurodegenerative disorders. Pharmacol Ther. 2021;219: 107705. 33039420 10.1016/j.pharmthera.2020.107705 PMC7887032

[158] Mosconi L, et al. Menopause impacts human brain structure, connectivity, energy metabolism, and amyloid-beta deposition. Sci Rep. 2021;11(1):10867. 34108509 10.1038/s41598-021-90084-y PMC8190071

[159] Klinge CM. Estrogens regulate life and death in mitochondria. J Bioenerg Biomembr. 2017;49(4):307–24. 28401437 10.1007/s10863-017-9704-1

[160] Yao J, et al. Ovarian hormone loss induces bioenergetic deficits and mitochondrial β-amyloid. Neurobiol Aging. 2012;33(8):1507–21. 21514693 10.1016/j.neurobiolaging.2011.03.001 PMC3181273

[161] Guglielmotto M, et al. Estrogens Inhibit Amyloid-β-Mediated Paired Helical Filament-Like Conformation of Tau Through Antioxidant Activity and miRNA 218 Regulation in hTau Mice. J Alzheimers Dis. 2020;77:1339–51. 32804095 10.3233/JAD-200707

[162] Guillot-Sestier M-V, et al. Microglial metabolism is a pivotal factor in sexual dimorphism in Alzheimer’s disease. Communications Biology. 2021;4(1):711. 34112929 10.1038/s42003-021-02259-y PMC8192523

[163] Munasinghe M, Ågren JA. When and why are mitochondria paternally inherited? Curr Opin Genet Dev. 2023;80: 102053. 37245242 10.1016/j.gde.2023.102053

[164] Mosconi L, et al. Maternal family history of Alzheimer’s disease predisposes to reduced brain glucose metabolism. Proc Natl Acad Sci. 2007;104(48):19067–72. 18003925 10.1073/pnas.0705036104 PMC2141909

[165] Berti V, et al. Structural brain changes in normal individuals with a maternal history of Alzheimer’s. Neurobiol Aging. 2011;32(12):2325.e17-2325.e26. 21316814 10.1016/j.neurobiolaging.2011.01.001 PMC3115436

[166] Honea RA, et al. Maternal Family History is Associated with Alzheimer’s Disease Biomarkers. J Alzheimers Dis. 2012;31:659–68. 22669011 10.3233/JAD-2012-120676 PMC3608420

[167] Mosconi L, et al. Oxidative stress and amyloid-beta pathology in normal individuals with a maternal history of Alzheimer’s. Biol Psychiatry. 2010;68(10):913–21. 20817151 10.1016/j.biopsych.2010.07.011 PMC2967599

[168] Lázaro I, et al. Omega-3 blood biomarkers relate to brain glucose uptake in individuals at risk of Alzheimer’s disease dementia. Alzheimer’s & Dementia: Diagnosis, Assessment & Disease Monitoring. 2024;16(3):e12596. 10.1002/dad2.12596 PMC11224768 38974876

[169] Freund-Levi Y, et al. ω-3 Fatty Acid Treatment in 174 Patients With Mild to Moderate Alzheimer Disease: OmegAD Study: A Randomized Double-blind Trial. Arch Neurol. 2006;63(10):1402–8. 17030655 10.1001/archneur.63.10.1402

[170] Huang Y, et al. Associations of fish oil supplementation with incident dementia: Evidence from the UK Biobank cohort study. Front Neurosci. 2022;16:910977. 36161159 10.3389/fnins.2022.910977 PMC9489907

[171] Wei B-Z, et al. The Relationship of Omega-3 Fatty Acids with Dementia and Cognitive Decline: Evidence from Prospective Cohort Studies of Supplementation, Dietary Intake, and Blood Markers. Am J Clin Nutr. 2023;117(6):1096–109. 37028557 10.1016/j.ajcnut.2023.04.001 PMC10447496

[172] Bischoff-Ferrari HA. et al. Individual and additive effects of vitamin D, omega-3 and exercise on DNA methylation clocks of biological aging in older adults from the DO-HEALTH trial. Nature Aging. 2025. 10.1038/s43587-024-00793-y PMC11922767 39900648

[173] Medeiros R, et al. Aspirin-triggered lipoxin A4 stimulates alternative activation of microglia and reduces Alzheimer disease-like pathology in mice. Am J Pathol. 2013;182(5):1780–9. 23506847 10.1016/j.ajpath.2013.01.051 PMC3644736

[174] Emre C, et al. Intranasal delivery of pro-resolving lipid mediators rescues memory and gamma oscillation impairment in AppNL-G-F/NL-G-F mice. Communications Biology. 2022;5(1):245. 35314851 10.1038/s42003-022-03169-3 PMC8938447

[175] Yin P, et al. Maresin 1 Improves Cognitive Decline and Ameliorates Inflammation in a Mouse Model of Alzheimer’s Disease. Front Cell Neurosci. 2019;13: 466. 31680874 10.3389/fncel.2019.00466 PMC6803487

[176] Singhaarachchi PH, et al. Aging, sex, metabolic and life experience factors: Contributions to neuro-inflammaging in Alzheimer’s disease research. Neurosci Biobehav Rev. 2024;162: 105724. 38762130 10.1016/j.neubiorev.2024.105724

[177] Wang X, et al. Resolution of inflammation is altered in Alzheimer’s disease. Alzheimers Dement. 2015;11(1):40-50.e2. 24530025 10.1016/j.jalz.2013.12.024 PMC4275415

[178] Do KV, et al. Cerebrospinal Fluid Profile of Lipid Mediators in Alzheimer’s Disease. Cell Mol Neurobiol. 2023;43(2):797–811. 35362880 10.1007/s10571-022-01216-5 PMC9957874

[179] Sang S, et al. Thiamine diphosphate reduction strongly correlates with brain glucose hypometabolism in Alzheimer’s disease, whereas amyloid deposition does not. Alzheimers Res Ther. 2018;10(1):26. 29490669 10.1186/s13195-018-0354-2 PMC5831864

[180] Jayaraman A, Pike CJ. Alzheimer’s Disease and Type 2 Diabetes: Multiple Mechanisms Contribute to Interactions. Curr DiabRep. 2014;14(4):476. 10.1007/s11892-014-0476-2 PMC3985543 24526623

[181] Carvalho C, Moreira PI. Metabolic defects shared by Alzheimer’s disease and diabetes: a focus on mitochondria. Curr Opin Neurobiol. 2023;79:102694. 36842275 10.1016/j.conb.2023.102694

[182] Norris PC, et al. Identification of specialized pro-resolving mediator clusters from healthy adults after intravenous low-dose endotoxin and omega-3 supplementation: a methodological validation. Sci Rep. 2018;8(1):18050. 30575798 10.1038/s41598-018-36679-4 PMC6303400

[183] Rathod KS, et al. Accelerated resolution of inflammation underlies sex differences in inflammatory responses in humans. J Clin Investig. 2023;127(1):169–81. 10.1172/JCI89429 PMC5199722 27893465

[184] Jové M, et al. Human aging is a metabolome-related matter of gender. Journals of Gerontology Series A: Biomedical Sciences and Medical Sciences. 2016;71(5):578–85. 10.1093/gerona/glv074 26019184

[185] Calder PC. Eicosapentaenoic and docosahexaenoic acid derived specialised pro-resolving mediators: Concentrations in humans and the effects of age, sex, disease and increased omega-3 fatty acid intake. Biochimie. 2020;178:105–23. 32860894 10.1016/j.biochi.2020.08.015

[186] Jónasdóttir HS, et al. Targeted lipidomics reveals activation of resolution pathways in knee osteoarthritis in humans. Osteoarthritis Cartilage. 2017;25(7):1150–60. 28189826 10.1016/j.joca.2017.01.018

[187] Kutzner L, et al. Development of an optimized LC-MS method for the detection of specialized pro-resolving mediators in biological samples. Front Pharmacol. 2019;10:169. 30899221 10.3389/fphar.2019.00169 PMC6416208

[188] O’Donnell VB, et al. Failure to apply standard limit-of-detection or limit-of-quantitation criteria to specialized pro-resolving mediator analysis incorrectly characterizes their presence in biological samples. Nat Commun. 2023;14(1):7172. 37945602 10.1038/s41467-023-41766-w PMC10636151

[189] Xavier S, et al. High maternal omega-3 supplementation dysregulates body weight and leptin in newborn male and female rats: implications for hypothalamic developmental programming. Nutrients. 2020;13(1):89. 33396616 10.3390/nu13010089 PMC7823471

[190] McEwen BJ, et al. Effects of omega-3 polyunsaturated fatty acids on platelet function in healthy subjects and subjects with cardiovascular disease. Semin Thromb Hemost. 2013;39(1):25–32. 23329646 10.1055/s-0032-1333309

[191] Naini AE, et al. Effect of Omega-3 fatty acids on blood pressure and serum lipids in continuous ambulatory peritoneal dialysis patients. J Res Pharm Pract. 2015;4(3):135–41. 26312252 10.4103/2279-042X.162356 PMC4548432

[192] Rabbani PI, et al. Subchronic toxicity of fish oil concentrates in male and female rats. J Nutr Sci Vitaminol (Tokyo). 2001;47(3):201–12. 11575575 10.3177/jnsv.47.201

[193] Pascoe MC, et al. Fish oil diet associated with acute reperfusion related hemorrhage, and with reduced stroke-related sickness behaviors and motor impairment. Front Neurol. 2014;5: 14. 24567728 10.3389/fneur.2014.00014 PMC3915239

[194] Morris MC, Sacks F, Rosner B. Does fish oil lower blood pressure? A meta-analysis of controlled trials Circulation. Circulation. 1993;88(2):523–33. 10.1161/01.cir.88.2.523 8339414

[195] Capucho AM, et al. Dysmetabolism and Neurodegeneration: Trick or Treat? Nutrients. 2022;14(7). 10.3390/nu14071425 PMC9003269 35406040

[196] Simopoulos AP. The importance of the ratio of omega-6/omega-3 essential fatty acids. Biomed Pharmacother. 2002;56(8):365–79. 12442909 10.1016/s0753-3322(02)00253-6

[197] Schuchardt JP, et al. Omega-3 world map: 2024 update. Prog Lipid Res. 2024;95: 101286. 38879135 10.1016/j.plipres.2024.101286

[198] Health, N.I.o. Omega-3 fatty acids: fact sheet for consumers. 2024 July 18, 2022; Available from: https://ods.od.nih.gov/factsheets/Omega3FattyAcids-Consumer/#:~:text=%2Dproducing%20glands).-,How%20much%20omega%2D3s%20do%20I%20need?,soy%20beverages%2C%20and%20infant%20formulas.

[199] Organisation, W.H. Healthy diet. 2020 29 April 2020. Available from: https://www.who.int/news-room/fact-sheets/detail/healthy-diet.

[200] Wang Y, et al. Pro-resolving lipid mediator reduces amyloid-β42-induced gene expression in human monocyte-derived microglia. Neural Regen Res. 2025;20(3):873–86. 38886959 10.4103/NRR.NRR-D-23-01688 PMC11433908

[201] Xian W, et al. Maresin 1 attenuates the inflammatory response and mitochondrial damage in mice with cerebral ischemia/reperfusion in a SIRT1-dependent manner. Brain Res. 2019;1711:83–90. 30639123 10.1016/j.brainres.2019.01.013

[202] West T, et al. A blood-based diagnostic test incorporating plasma Aβ42/40 ratio, ApoE proteotype, and age accurately identifies brain amyloid status: findings from a multi cohort validity analysis. Mol Neurodegener. 2021;16(1):30. 33933117 10.1186/s13024-021-00451-6 PMC8088704

[203] Cullen NC, et al. Test-retest variability of plasma biomarkers in Alzheimer's disease and its effects on clinical prediction models. Alzheimers Dement. 2022. 10.1002/alz.12706 PMC9747985 35699240

[204] González-Domínguez R, García-Barrera T, Gómez-Ariza JL. Metabolomic study of lipids in serum for biomarker discovery in Alzheimer’s disease using direct infusion mass spectrometry. J Pharm Biomed Anal. 2014;98:321–6. 24992214 10.1016/j.jpba.2014.05.023

[205] Fairley LH, et al. Mitochondrial control of microglial phagocytosis by the translocator protein and hexokinase 2 in Alzheimer’s disease. Proc Natl Acad Sci U S A. 2023;120(8): e2209177120. 36787364 10.1073/pnas.2209177120 PMC9974442

[206] Kreisl WC, et al. In vivo radioligand binding to translocator protein correlates with severity of Alzheimer’s disease. Brain. 2013;136(Pt 7):2228–38. 23775979 10.1093/brain/awt145 PMC3692038

[207] Jurga AM, Paleczna M, Kuter KZ. Overview of General and Discriminating Markers of Differential Microglia Phenotypes. Front Cell Neurosci. 2020;14:198. 32848611 10.3389/fncel.2020.00198 PMC7424058

[208] Hwangbo N, et al. Predictive Modeling of Alzheimer's and Parkinson's Disease Using Metabolomic and Lipidomic Profiles from Cerebrospinal Fluid. Metabolites. 2022;12(4). 10.3390/metabo12040277 PMC9029812 35448464

